# Supplementary material for: Bigger Data Approach to Analysis of Essential Oils and Their Antifungal Activity against Aspergillus niger, Candida albicans, and Cryptococcus neoformans
Source: Molecules. 2019 Aug 7;24(16):2868. doi: 10.3390/molecules24162868 (PMC6718987; doi:10.3390/molecules24162868)
Supplement: Supplementary file 1 [file molecules-24-02868-s001.pdf]

Supplementary Figure 1. Tables of GC-MS data for each of the 82 essential oils reported herein.

1.) Ambrette Seed

RT	Area%	Component	RI
37.263	0.12	Carvacrol	1296
44.644	1.56	Decyl acetate	1408
47.389	0.05	Farnesene <E,beta>	1452
55.59	0.23	Hexadecene	1587
56.785	2.07	Dodecyl acetate	1607
61.487	0.05	Farnesal <2Z,6Z>	1690
62.425	0.05	Farnesal <2E,6Z>	1707
62.758	0.66	Farnesol <2E,6Z>	1713
63.353	0.46	Oxacyclopentadec-6-en-2-one <Z>	1725
63.918	0.1	Farnesal <2E,6E>	1735
66.004	0.14	9,12-Tetradecadien-1-ol, acetate <Z,Z>	1774
66.504	0.76	cis-7-Tetradecen-1-yl acetate	1783
67.743	4.33	Farnesyl acetate <Z,E>	1806
69.106	59.65	Farnesyl acetate <E,E>	1833
73.524	0.73	Farnesyl acetone <5E,9E>	1920
73.738	0.12	Palmitate <methyl>	1924
74.229	8.89	Oxacycloheptadec-7-en-2-one	1934
74.397	0.11	Beyerene	1938
75.431	3.87	Palmitic acid	1959
77.7	0.34	Geranyl linalool <Z,E>	2006
79.663	0.12	Geranyl linalool <E,E>	2048
81.654	0.1	Methyl linoleate	2090
81.998	0.06	Methyl octadecanoate <9E>	2098
83.39	5.65	Linoleic acid <Z>	2128
83.701	8.56	Oleic Acid	2135
84.736	0.64	Octadecanoic acid	2158
85.016	0.11	Ethyl oleate	2165
86.56	0.06	Docosane	2199
103.091	0.09	Docosyl acetate	2608

2.) Yarrow

RT	Area %	Component	RI
12.547	0.29	Thujene <alpha>	925
12.973	1.72	Pinene <alpha>	932
15.219	17.18	Sabinene	972
15.534	11	Pinene <beta>	977
16.145	0.5	Myrcene	988
17.918	0.35	Terpinene <alpha>	1017
18.407	0.28	Cymene <para>	1024
18.715	0.52	Limonene	1029
18.933	3.42	1,8-cineole	1032
19.81	0.39	Ocimene <E,beta>	1045
20.574	6.18	Artemisia ketone	1056
23.43	2.63	Linalool	1099
26.763	0.33	Camphor	1147
27.87	0.37	Artemisyl acetate	1163
29.088	3.49	Terpinen-4-ol	1180
30.088	0.68	Terpineol <alpha>	1195
33.824	2.04	Linalyl acetate	1249
35.188	0.97	4-Thujen-2-alpha-yl acetate	1269
35.677	0.31	Ethyl chrysenthmumate	1276
36.134	1.12	Lavandulyl acetate	1282
36.224	0.51	Bornyl acetate	1284
42.835	0.56	Bourbonene <beta>	1383
45.155	15	Caryophyllene <E>	1419
47.205	0.89	Farnesene <E,beta>	1452
47.396	1.79	Humulene <alpha>	1455
49.01	19.06	Germacrene D	1481
49.855	1.42	Farnesene <Z,E,alpha>	1494
51.244	0.42	Cadinene <delta>	1517
55.06	1.93	Caryophyllene oxide	1581
63.319	4.65	Chamazulene	1727

3.) Fragonia

RT	Area %	Component	RI
10.402	0.25	Thujene <alpha>	925
10.801	22.7	Pinene <alpha>	932
12.826	0.35	Sabinene	971
13.102	1.72	Pinene <beta>	976
13.74	1.05	Myrcene	989
15.37	0.12	Terpinene <alpha>	1016
15.846	5.15	Cymene <para>	1024
16.133	2.68	Limonene	1029
16.35	37.2	1,8-cineole	1032
17.939	0.6	Terpinene <gamma>	1057
20.693	7.77	Linalool	1100
23.355	0.3	Pinocarveol <trans>	1139
25.395	0.33	Terpineol <delta>	1169
26.094	3.62	Terpinen-4-ol	1179
27.143	10.73	Terpineol <alpha>	1195
29.085	0.33	Nerol	1223
29.276	0.5	Citronellol	1226
30.859	0.88	Geraniol	1250
31.665	0.35	Myrtanol <trans>	1261
35.675	0.86	Myrtenyl acetate <Z>	1321
41.829	0.32	Caryophyllene <trans>	1416
43.003	0.21	Aromadendrene	1435
44.076	0.5	Humulene <alpha>	1452
46.284	0.17	Viridiflorene	1487
48.159	0.13	Calamenene <trans>	1518
51.384	0.37	Spathulenol	1572
51.91	0.19	Globulol	1581
52.414	0.15	Viridifloral	1590
54.623	0.2	Acorenol	1628
55.88	0.27	Eudesmol <alpha>	1651

4.) Ajowan

RT	Area %	Component	RI
11.314	0.07	Thujene <alpha>	923
11.703	0.71	Pinene <alpha>	931
14.101	2	Pinene <beta>	977
14.732	0.59	Myrcene	988
15.761	0.1	Phellandrene <alpha>	1004
15.891	0.04	Carene <delta-3>	1006
16.403	0.42	Terpienene <alpha>	1014
16.9	16.32	Cymene <para>	1022
17.165	0.42	Limonene	1026
17.268	0.38	Phellandrene <beta>	1028
19.065	45.08	Terpinene <gamma>	1056
19.8	0.04	Sabinene hydrate <cis>	1067
20.772	0.08	Terpinolene	1083
21.108	0.11	Cymenene <para>	1088
22.699	0.09	2,4-dimethyl-heptadienal	1111
24.477	0.06	Menthatriene <1,3,8-para>	1137
25.144	0.06	Verbenol	1147
26.069	0.04	Mentha-2,8-dien-1-ol <trans-para>	1160
26.315	0.14	Menthol	1164
27.112	0.1	1,8-menthadien-4-ol <para>	1176
27.243	0.23	Terpinen-4-ol	1178
27.705	0.13	Cymen-8-ol <para>	1184
28.265	0.12	Terpineol <alpha>	1193
28.464	0.07	Carvone <dihydro-trans>	1196
31.601	0.09	Carvone	1242
34.122	0.22	Isothymol	1279
34.786	31.68	Thymol	1289
35.246	0.43	Carvacrol	1296
37.263	0.06	Mentha-1,4,-dien-7-ol <para>	1327
55.046	0.12	Dill apiole	1618

5.) Getto

RT	Area%	Component	RI
12.641	4.91	Thujene <alpha>	925
13.068	2.33	Pinene <alpha>	932
14.009	0.23	Camphene	949
15.319	13.71	Sabinene	972
15.634	5.62	Pinene <beta>	977
16.252	1.1	Myrcene	988
17.356	0.3	Phellandrene <alpha>	1006
18.033	2.75	Terpinene <alpha>	1016
18.538	13.17	Cymene <para>	1024
18.837	2.45	Limonene	1028
18.95	0.62	Phellandrene <beta>	1030
19.07	17.27	1,8-cineole	1032
20.763	14.88	Terpinene <gamma>	1057
21.561	0.17	4-Thujanol <cis>	1069
22.583	1.72	Terpinolene	1084
23.544	0.62	Linalool	1099
23.665	0.16	Sabinene hydrate <trans>	1101
25.289	0.34	Menth-2-en-1-ol <cis,p>	1124
26.536	0.29	Menth-2-en-1-ol <trans,p>	1142
29.235	12.45	Terpinen-4-ol	1180
30.21	0.61	Terpineol <alpha>	1194
31.163	0.12	Piperitol <trans>	1208
35.635	0.29	Ascaridol glycol <E>	1273
45.269	1.3	Caryophyllene <trans>	1419
51.077	0.16	Amorphene <delta>	1512
53.92	0.2	Nerolidol <trans>	1560
55.201	1.48	Caryophyllene oxide	1581

6.) Amyris

RT	Area%	Component	RI
46.991	0.29	Amorpha-4,11-diene	1448
47.493	0.98	Himachalene <alpha>	1456
48.754	0.8	Curcumene <gamma>	1477
48.954	1.45	Curcumene	1480
49.688	0.3	Selinene <beta>	1491
49.821	2.26	Zingiberene <alpha>	1494
50.5	1.03	Dihydroagarofuran <beta>	1505
50.615	0.86	Bisabolene <beta>	1506
51.317	0.28	Selinene <7-epi,alpha>	1518
51.58	3.82	Sesquiphellandrene <beta>	1523
52.651	0.71	Selina-3,7(11)-diene	1541
53.04	11.55	Elemol <alpha>	1547
53.775	0.49	Nerolidol <E>	1560
56.489	1.65	Eudesmol <5-epi,7-epi,alpha>	1605
56.913	0.16	Zingiberenol	1613
57.412	8	Eudesmol <10-epi,gamma>	1622
57.54	1.54	Eremoligenol	1624
57.936	7.01	Eudesmol <gamma>	1631
58.399	0.19	Hinesol	1639
58.66	0.57	Agarospirol II	1644
58.761	0.59	Sesquiterpene	1645
59.214	39.05	Eudesmol <alpha>	1653
59.614	12.2	Eudesmol <7-epi,alpha>	1661
60.616	1.06	Germacra-4(15),5,10(14)-trien-1-alpha-ol	1678
61.584	0.56	Farnesol <2Z,6Z>	1695
64.042	0.56	Bisabolone <6S,7R>	1741
64.581	0.28	Bisabolone	1751
65.497	1.76	Drimenol	1768

7.) Agarwood

RT	Area%	Component	RI
14.538	0.63	Benzaldehyde	960
33.248	5.07	Benzylacetone	1240
50.244	0.98	Bulnesene <alpha>	1500
53.925	2.51	Calcorene <alpha>	1562
54.752	1.2	Caryophyllene oxide	1576
55.56	1.96	Sesquithuriferol	1589
56.714	0.75	Atlantol <beta>	1609
57.39	3.35	Eremoligenol	1621
57.92	3.33	Eudesmol <gamma>	1631
58.164	3.34	Hinesol	1635
58.37	4.61	Agarospirol	1639
58.464	6.25	Alloaromadendrene epoxide	1640
59.157	6.48	Valerianol	1652
59.234	9.58	alpha-Eudesmol	1654
59.387	2.82	Cadinol <alpha>	1656
59.785	2.01	Bulnesol	1664
60.262	8.36	Guaia-3,9-dien-11-ol <trans>	1672
61.395	0.89	Norketoagarofuran	1692
61.603	5.92	10-alpha-Eremophilane	1696
62.01	1.61	Mayurone	1703
63.594	2.09	Eremophilone	1732
64.316	2.12	Zerumbone	1746
67.247	4.48	Eremophila-7(11),9-dien-8-one	1800
74.691	2.36	Columellarin	1948

8.) Tarragon

RT	Area %	Component	RI
11.686	0.96	Pinene <alpha>	930
12.56	0.05	Camphene	947
13.792	0.08	Sabinene	971
14.081	0.12	Pinene <beta>	976
14.717	0.12	Myrcene	987
15.717	0.03	Hexenyl acetate	1003
16.873	0.02	Terpinene <alpha>	1021
17.144	2.7	Limonene	1026
17.56	6.14	Ocimene <Z,beta>	1032
18.228	6.33	Ocimene <E,beta>	1043
20.016	0.06	Pinene oxide <alpha>	1071
21.428	0.05	Pinene oxide <beta>	1092
21.738	0.04	Linalool	1097
23.629	0.24	Ocimene	1125
23.755	0.03	Epoxy ocimene <cis>	1126
24.415	0.06	Epoxy ocimene <trans>	1136
28.565	81.75	Methyl chavicol	1197
32.101	0.02	Tarragon	1249
33.237	0.1	Decen-1-ol <2E>	1266
34.258	0.09	Bornyl acetate	1281
38.686	0.2	Eugenol	1349
40.862	0.09	Methyl cinnamate <E>	1382
41.88	0.34	Methyleugenol	1398
43.03	0.02	Caryophyllene <trans>	1417
46.865	0.04	Germacrene D	1478
47.553	0.04	Farnesene	1490
49.254	0.03	Geranyl isobutanoate	1518
52.2	0.03	Cinnamaldehyde <E,para-methoxy>	1568
52.58	0.19	Spathulenol	1574
60.775	0.03	Hemianin	1721

9.) Davana

RT	Area%	Component	RI
16.858	0.3	Cymene <para>	1021
21.727	0.38	Linalool	1097
21.954	0.33	2-methylbutyrate	1100
37.398	0.45	Elemene <delta>	1329
40.24	0.98	Ethyl cinnamate <cis>	1372
40.49	0.71	Geranyl acetate	1376
40.835	0.55	Methyl cinnamate <E>	1382
45.555	0.48	Alloaromadendrene	1457
46.072	4.35	Ethyl cinnamate <E>	1465
46.88	0.82	Germacrene D	1478
47.105	1.28	Davana ether 1	1482
47.371	1.25	Selinene <beta>	1487
47.49	0.37	Viridiflorene	1489
47.782	8.45	Bicyclogermacrene	1493
48.294	4.3	Davana ether 2	1502
48.385	1.03	Davana ether 3	1504
48.846	0.25	Amorphene <delta>	1511
49.448	2.6	Davana ether 4	1522
49.764	0.29	Artedouglasia oxide A	1527
51.081	0.51	Davanone B	1549
51.481	1.61	Davanone C	1556
51.803	0.6	Nerolidol <trans>	1561
52.6	0.87	Spathulenol	1575
52.874	63.41	Davanone	1579
56.382	1.25	Cadinol <epi,alpha>	1641
57.08	0.74	Eudesmol <alpha>	1654

10.) Mugwort

RT	Area %	Component	RI
11.221	3.46	Santolina triene	901
12.945	4.42	Pinene <alpha>	932
13.78	2.95	Fenchene <alpha>	947
13.879	0.82	Camphene	948
15.172	3.78	Sabinene	971
15.494	10.96	Pinene <beta>	977
16.44	1.24	Yomogi alcohol	993
18.372	1.68	Cymene <para>	1023
18.678	1.24	Limonene	1028
18.892	9.41	1,8-cineole	1031
19.771	0.74	Ocimene <E,beta>	1044
22.009	0.91	Artemisia alcohol	1078
23.451	2.49	Filifolone	1100
23.894	5.9	Thujone <cis>	1106
24.526	4.42	Chrysanthenol <trans>	1115
24.67	1.59	Thujone <trans>	1117
24.911	1.53	Chrysanthenone	1120
25.95	2.18	4,5-epoxy-carene <E>	1135
26.332	1.14	Eucarvone	1141
26.715	1.13	Camphor	1146
29.033	1.13	Terpinen-4-ol	1179
30.051	1.25	Terpineol <alpha>	1194
44.293	0.91	Gurjunene <alpha>	1406
45.089	12.07	Caryophyllene <E>	1418
47.144	1.14	Farnesene <E,beta>	1451
47.34	1.63	Humulene <alpha>	1454
48.933	8.27	Germacrene D	1479
51.19	1.08	Cadinene <delta>	1516
51.532	1.77	Sesquiphellandrene <beta>	1522
54.755	8.76	Davanone	1576

11.) Palo Santo

RT	Area %	Component	RI
12.977	0.11	Pinene <alpha>	932
15.888	0.12	Octen-2-ol <3E>	984
15.969	0.14	Acetylcyclohexane	985
16.147	0.3	Myrcene	988
16.242	0.18	1,8-cineole <dehydro>	990
18.417	0.77	Cymene <para>	1024
18.807	70.7	Limonene	1030
18.885	0.11	Phellandrene <beta>	1031
18.962	0.15	1,8-cineole	1032
24.969	0.26	Mentha-2,8-dien-1-ol <trans,para>	1121
25.985	0.34	Mentha-2,8-dien-1-ol <cis,para>	1136
26.894	0.13	Terpineol <cis,beta>	1149
27.366	0.38	Menthone	1156
27.898	12.87	Menthofuran	1163
28.93	0.21	1,8-menthadien-4-ol <para>	1178
30.11	7.87	Terpineol <alpha>	1195
30.291	0.23	Dihydro carvone <cis>	1197
30.425	0.28	Piperitol <Z>	1199
31.706	0.39	Carveol <trans>	1218
32.619	0.14	Carveol <cis>	1231
33.044	0.92	Pulegone	1237
33.428	0.82	Carvone	1243
43.184	0.19	Elemene <beta>	1388
46.991	0.28	Myltayl-4(12)-ene	1448
48.588	0.4	Cadina-1(6),4-diene <trans>	1474
48.977	1.1	Germacrene D	1480
49.854	0.24	Menthalactone	1494
50.391	0.16	Farnesene <E,E,alpha>	1503
51.235	0.21	Cadinene <delta>	1517

12.) Blue Cypress

RT	Area %	Component	RI
11.817	0.27	Pinene <alpha>	933
14.625	0.4	Hept-5-en-2-one <6-methyl>	986
36.228	0.39	Citronellic acid	1311
36.949	1.92	Geranate <methyl>	1322
41.355	0.33	Elemene <beta>	1390
42.901	0.7	Jasmine lactone <cis>	1415
46.581	3.01	Neocallitropsene <alpha>	1474
47.398	0.48	Dihydroagarofuran	1487
47.572	5.22	Selinene <beta>	1490
48.005	4.17	Selinene <alpha>	1497
51.143	1.32	Elemol <alpha>	1550
54.011	13.36	Guaiol	1599
54.913	0.49	Eudesmol <5-epi,7-epi,beta>	1615
56.008	10.28	gamma-Eudesmol	1635
56.447	0.32	Hinesol	1642
57.331	25.02	Eudesmol <alpha>	1658
57.485	1.89	Selin-11-en-4-alpha-ol	1661
57.892	20.47	Bulnesol	1668
64.016	1.37	Methyl aciphyllate	1781
66.069	0.61	Callitrin	1822
68.16	0.29	Callistrin A	1862
70.175	1.67	Dihydrocostunilide	1903
72.572	0.46	Columellarin	1952

13.) Caraway

RT	Area %	Component	RI
11.15	0.03	Heptanal	901
15.095	0.02	Sabinene	971
16.025	0.13	Myrcene	987
16.905	0.04	n-Octanal	1002
18.555	26.58	Limonene	1028
23.31	0.04	Linalool	1098
24.84	0.1	Mentha-2,8-dien-1-ol <trans,para>	1120
25.85	0.12	Mentha-2,8-dien-1-ol <cis,para>	1135
27.5	0.02	Nonen-1-al <2E>	1158
27.8	0.07	Cyclohexadiene	1163
29.435	0.04	Mentha-1(7),8-dien-2-ol <trans,p>	1186
29.945	0.17	Terpineol <alpha>	1194
30.14	0.43	Dihydro carvone <cis>	1197
30.3	0.09	Piperitol <cis>	1198
30.595	0.18	Dihydro carvone <trans>	1203
31.51	0.13	Dihydrocarveol	1216
31.62	0.22	Carveol <trans>	1217
32.33	0.03	Carveol <cis>	1228
32.445	0.62	Dihydro carveol	1230
33.255	70.34	Carvone	1244
35.46	0.2	Perilla aldehyde	1274
38.565	0.02	Geranate <methyl>	1319
39.31	0.03	Carvyl acetate <trans>	1331
44.98	0.11	Caryophyllene <trans>	1418
47.065	0.07	Farnesene <E,beta>	1451
48.875	0.02	Germacrene D	1479
51.49	0.08	Dehydro Lachnophyllum ester	1523
54.93	0.07	Caryophyllene oxide	1580

14.) Katrafay

RT	Area %	Component	RI
15.653	0.95	Pinene <beta>	977
42.003	1.19	Isoledene	1368
42.472	9.2	Copaene <alpha>	1375
43.357	8.52	Elemene <beta>	1388
44.19	1.06	Sesquithujene	1401
44.259	1.23	Cyperene	1402
45.299	0.7	Caryophyllene <trans>	1419
46.956	3.69	Myltayl-4(12)-ene	1445
47.413	1.13	Farnesene <E,beta>	1452
47.593	10.81	Cedrolopsis	1455
47.839	2.32	(-)-Alloaromadendrene	1459
47.959	1.84	Rotundene	1461
48.082	0.67	Acoradiene <alpha>	1463
48.439	29.5	Ishwarene	1469
48.732	2.45	Caryophyllene <9-epi>	1473
48.932	0.84	Curcumene <gamma>	1477
49.132	5.2	Curcumene	1480
49.376	2.01	Aristolochene	1484
49.65	1.15	Selinene <beta>	1488
49.856	0.73	Valencene	1491
50.129	3.51	Guaine <Z,beta>	1496
50.231	1.52	Muurolene <alpha>	1497
50.789	1.24	Bisabolene <beta>	1506
50.896	1.26	Curcumene <beta>	1508
51.124	1.09	Sesquicineole	1512
51.408	2.43	Cadinene <delta>	1517
51.609	2.03	Calamenene <trans>	1520
55.23	0.92	Caryophyllene oxide <beta>	1581
61.232	0.81	Bisabolol <alpha>	1686

15.) Atlas Cedarwood (1)

RT	Area %	Component	RI
27.924	1.09	4-Acetyl-1-methylcyclohexene	1136
44.764	0.32	Isolongofolene <4,5-dehydro>	1391
46.121	0.59	Gurjunene <alpha>	1413
46.755	0.52	Caryophyllene <9-epi,cis>	1423
47.466	0.38	6,9-guaiadiene	1434
48.354	0.49	Vestitenone	1449
49.35	16.12	Caryophyllene <9-epi>	1465
49.757	0.21	Cedarwood	1472
50.441	0.33	Himachalene <gamma>	1483
51.006	10.74	Viridiflorene	1492
51.24	2.33	Himachalene <beta>	1496
52.443	42.62	Curcumene <beta>	1516
52.699	0.99	Himachalene <alpha,dehydro>	1520
53.053	2.59	Cadinene <delta>	1526
53.691	2.54	Himachalene <gamma,dehydro>	1537
54.131	1.49	Bisabolene <trans,alpha>	1545
54.5	1.03	Calacorene <alpha>	1551
56.341	0.54	Himachalene epoxide	1582
58.336	0.31	Ledol	1617
58.78	0.5	Epi-Cedrol	1625
59.496	0.74	Cubenol <1-epi>	1638
59.744	0.17	Himachalol <alpha>	1643
61.166	1.08	Bisabolol <6-epi,alpha>	1668
61.845	1.04	Cedranol <5-neo>	1680
62.539	1.04	Atlantone <Z,gamma>	1693
63.195	2.62	Atlantone <E,gamma>	1705
63.906	0.93	Atlantone <Z,alpha>	1718
67.234	4.62	Atlantone <E,alpha>	1781

16.) Atlas Cedarwood (2)

RT	Area%	Component	RI
12.937	0.17	Pinene <alpha>	932
42.683	0.69	Funebrene <2-epi,alpha>	1381
42.964	0.88	Duprezianene <alpha>	1385
43.866	0.19	Longipinene <beta>	1399
43.997	0.21	Chamipinene <alpha>	1401
44.912	31.4	Cedrene <alpha>	1415
45.087	1.55	Caryophyllene <E>	1418
45.385	6.08	Funberene <beta>	1423
46.094	20.45	Thujopsene <cis>	1434
46.23	0.16	Isobazzanene	1436
47.099	0.21	Prezizaene	1450
47.854	0.17	Acoradiene <alpha>	1462
48	0.42	Acoradiene <beta>	1465
48.948	1.12	Gurjunene <gamma>	1480
50.311	1.77	Himachalene <beta>	1501
50.377	1.55	Pseudowiddrene	1502
50.486	0.66	Chamigrene <alpha>	1504
50.619	1.14	Cuparene	1507
51.178	0.32	Alaskene	1516
52.181	1.74	Cuprenene <gamma>	1533
52.572	0.23	Liguloxide	1539
52.939	0.24	Cuprenene <delta>	1545
56.478	3.25	Widdrol	1605
56.615	23.52	Cedrol	1608
57.386	0.31	Cedrol <epi>	1621
58.48	0.36	Sesquiterpineol	1640
59.252	0.28	Thujopsanone <3>	1654
59.653	0.25	Cedr-8-en-15-ol	1661
60.875	0.38	Bisabolol <alpha>	1683
62.119	0.3	Thujopsenal <cis>	1705

17.) Hinoki Cypress

RT	Area%	Component	RI
13.008	42.62	Pinene <alpha>	933
15.208	1.72	Sabinene	972
15.526	0.48	Pinene <beta>	977
16.148	1.49	Myrcene	988
17.922	0.28	Terpinene <alpha>	1017
18.72	1.93	Limonene	1029
20.628	0.71	Terpinene <gamma>	1057
22.469	0.6	Terpinolene	1085
29.082	0.51	Terpinen-4-ol	1180
30.086	0.46	Terpineol <alpha>	1195
36.221	0.98	Bornyl acetate	1284
40.355	2.13	Terpinyl acetate <alpha>	1346
43.19	1.55	Elemene <beta>	1389
47.792	0.31	Muurola-4(14),5-diene <cis>	1461
48.413	0.42	Cadina-1(6),4-diene <cis>	1471
48.593	2.07	Cadina-1(6),4-diene <trans>	1474
48.983	0.47	Germacrene D	1480
49.481	0.4	Selinene <beta>	1488
49.916	0.61	Valencene	1495
50.057	3.1	Muurolene <alpha>	1497
50.946	5.14	Amorphene <delta>	1512
51.254	9.69	Cadinene <delta>	1517
52.347	0.61	Cadinene <alpha>	1536
53.026	0.34	Elemol	1547
57.697	0.65	Cubenol <1-epi>	1627
58.503	4.11	Muurolol <alpha>	1641
58.617	5.49	Murrolol <epi,alpha>	1643
58.763	1.84	Cadinol <delta>	1645
59.265	8.88	Cadinol <alpha>	1654
59.436	0.41	Selin-11-en-4-alpha-ol	1657

18.) Chamomile

RT	Area%	Component	RI
9.781	1.94	Isobutyl isobutyrate	913
10.765	3.97	Pinene <alpha>	932
10.966	2.88	Methacrylic acid, isobutyl ester	936
11.605	1.19	Camphene	948
11.729	1.19	Fruitaleur	950
13.873	0.52	Ethyl tiglate	991
14.486	0.63	Isobutyl 2-methylbutanoate	1003
15.084	0.51	Isoamyl isobutyrate	1012
15.277	3.16	2-Methylbutyl isobutyrate	1015
16.805	1.7	Isopentyl methacrylate	1039
16.881	3.37	Prenyl	1040
17.441	14.84	Isobutyl angelate	1049
18.305	8.75	Methallyl angelate	1063
19.57	0.34	Camphenilone	1083
19.904	0.46	Butyl angelate	1088
20.885	0.81	Butyrate <2-methyl,3-methylbutyl>	1103
21.731	1.26	Hexyl isobutyrate	1115
23.368	4.39	Pinocarveol <trans>	1139
23.676	3.8	Butanoic acid <ester>	1144
23.892	7.61	Isoamyl angelate	1147
24.178	16.9	Isoamyl tiglate	1151
24.345	0.6	Terpineol <E,beta>	1154
24.839	2.18	Pinocarvone	1161
25.489	0.65	Borneol	1171
26.819	1.26	Angelate <2-methyl-2-butene>	1190
27.088	0.59	Myrtenol	1194
30.086	0.37	2-methyl-2-butenoate <2E>	1238
30.95	11.42	3-methyl-pentylangelate	1251
45.67	2.32	Germacrene D	1477
47.243	0.39	Farnesene <E,E,alpha>	1503

19.) Wild Vetiver

RT	Area%	Component	RI
47.102	0.9	Prezizaene	1450
47.394	0.92	Khusimene	1455
53.322	0.78	Vetivenene <beta>	1552
55.004	0.57	Caryophyllene oxide	1580
56.356	7.47	Khusimone	1603
56.908	1.53	Farnesol <2Z,6Z>	1613
57.36	1.08	Selina-6-en-4-ol	1621
58.156	0.94	Caryophylla-4(12),8(13)-dien-5 beta-ol	1635
58.547	1.13	Cedrenal <1,7-diepi,alpha>	1642
58.681	4.41	Khusilal	1644
59.136	2.66	Cadinol <alpha>	1652
59.321	1.45	Intermedeol	1655
59.475	0.8	Himachalol	1658
59.74	0.61	Vetiver	1663
59.834	2.21	Caryophyllene <14-hydroxy,Z>	1664
60.056	10.64	Caryophyllene <14-hydroxy,9-epi,E>	1668
60.338	5.13	Khusinol	1673
60.429	1.8	Uvetiver	1675
60.975	1.43	Zizanone <epi>	1685
61.079	5.03	Nootkatol	1686
61.378	4.83	Acoranol	1692
61.604	2.28	Acorenone B	1696
61.736	3	Amorphadienol	1698
64.222	26.38	Khusimol	1744
64.445	1.29	Cyclocolorenone	1748
64.974	0.95	Curcumen-12-ol <beta,Z>	1758
67.249	9.78	Vetivenic acid <cis>	1800

20.) Ravintsara

RT	Area%	Component	RI
12.529	0.84	Thujene <alpha>	925
12.958	4.72	Pinene <alpha>	932
13.892	0.17	Camphene	948
15.198	12.79	Sabinene	972
15.507	3.3	Pinene <beta>	977
16.125	1.39	Myrcene	988
17.226	0.06	Phellandrene <alpha>	1006
17.898	0.88	Terpinene <alpha>	1016
18.394	0.38	Cymene <para>	1024
18.71	0.84	Limonene	1028
18.974	59.87	1,8-cineole	1032
19.088	0.07	Ocimene <Z,beta>	1034
19.788	0.31	Ocimene <E,beta>	1045
20.605	1.38	Terpinene <gamma>	1057
21.413	0.48	Sabinene hydrate <cis>	1069
22.441	0.35	Terpinolene	1084
23.399	0.04	Linalool	1099
23.512	0.33	Sabinene hydrate <trans>	1100
25.14	0.08	Menth-2-en-1-ol <cis,para>	1124
26.382	0.04	Menth-2-en-1ol <trans,para>	1141
28.328	0.52	Terpineol <delta>	1169
28.462	0.1	Borneol	1171
29.058	2.24	Terpinen-4-ol	1180
30.079	6.93	Terpineol <alpha>	1194
43.158	0.08	Elemene <beta>	1388
45.105	0.55	Caryophyllene <E>	1418
47.363	0.7	Humulene <alpha>	1454
48.952	0.19	Germacrene D	1480
49.451	0.08	Selinene <beta>	1488
49.854	0.29	Bicyclogermacrene	1494

21.) Sugandha Kokila

RT	Area %	Component	RI
11.302	0.59	Thujene <alpha>	923
11.693	3.51	Pinene <alpha>	931
12.565	0.1	Camphene	947
13.8	3.64	Sabinene	971
14.091	4.35	Pinene <beta>	976
14.72	1.83	Myrcene	987
15.758	12.53	Phellandrene <alpha>	1004
16.39	1.37	Terpinene <alpha>	1014
16.871	3.6	Cymene <para>	1021
17.161	1.87	Limonene	1026
17.268	8.46	Phellandrene <beta>	1028
17.361	20.61	1,8-cineole	1029
18.221	0.44	Ocimene <E,beta>	1042
18.984	1.82	Terpinene <gamma>	1055
20.756	0.42	Terpinolene	1082
21.736	2.48	Linalool	1097
24.926	1.76	Camphor	1144
27.227	2.78	Terpinen-4-ol	1177
28.242	4.08	Terpineol <alpha>	1192
33.187	0.35	Geranial	1265
40.261	1.34	Copaene <alpha>	1373
40.879	12.37	Methyl-cinnamate <E>	1382
41.895	1.21	Methyleugenol	1399
43.047	2.47	Caryophyllene <trans>	1417
47.374	0.33	Selinene <beta>	1487
47.816	0.65	Methyl isoeugenol <E>	1494
48.585	0.38	Bisabolene <beta>	1507
49.171	1.03	Cadinene <delta>	1517
49.299	3.34	Myristicin	1519
50.924	0.29	Elemicin	1546

22.) Neroli

RT	Area%	Component	RI
12.937	0.26	Pinene <alpha>	932
15.163	0.49	Sabinene	971
15.483	3.88	Pinene <beta>	977
16.102	1.38	Myrcene	987
17.874	0.04	Terpinene <alpha>	1016
18.364	0.03	Cymene <para>	1023
18.678	9.18	Limonene	1028
18.785	0.05	Phellandrene <beta>	1030
19.063	0.78	Ocimene <Z,beta>	1034
19.764	3.8	Ocimene <E,beta>	1044
20.576	0.07	Terpinene <gamma>	1056
21.408	0.09	Linalool oxide <cis>	1069
22.415	0.33	Terpinolene	1084
22.505	0.06	Linalool oxide <trans>	1085
23.438	39.44	Linalool	1099
25.951	0.05	Benzeneacetonitrile	1135
29.023	0.12	Terpinen-4-ol	1179
30.031	3.31	Terpineol <alpha>	1194
32.007	0.64	Nerol	1222
33.793	26.71	Linalyl acetate	1248
34.058	0.21	2-phenyl ethyl acetate	1252
36.515	0.05	Indole	1288
39.809	0.17	Anthranilate <methyl>	1337
40.293	0.1	Terpinyl acetate <alpha>	1345
41.059	1.76	Neryl acetate	1356
42.351	3.48	Geranyl acetate	1376
45.066	0.5	Caryophyllene <E>	1418
49.808	0.11	Bicyclogermacrene	1493
53.712	1.7	Nerolidol <E>	1558
62.476	1.21	Farnesol <2E,6Z>	1712

23.) Petitgrain

RT	Area%	Component	RI
12.531	0.02	Thujene <alpha>	925
12.955	0.12	Pinene <alpha>	932
15.183	0.2	Sabinene	971
15.501	0.83	Pinene <beta>	977
16.123	1.75	Myrcene	988
17.222	0.04	Phellandrene <alpha>	1006
17.372	0.34	Carene <delta-3>	1008
17.897	0.04	Terpinene <alpha>	1016
18.69	0.75	Limonene	1028
18.802	0.04	Phellandrene <beta>	1030
18.909	0.08	1,8-cineole	1031
19.085	0.64	Ocimene <Z,beta>	1034
19.784	1.89	Ocimene <E,beta>	1045
20.604	0.07	Terpinene <gamma>	1057
21.436	0.04	Linalool oxide <cis>	1069
22.437	0.38	Terpinolene	1084
23.417	21.09	Linalool	1099
29.051	0.09	Terpinen-4-ol	1180
30.056	4.64	Terpineol <alpha>	1194
32.037	0.72	Nerol	1223
33.824	59.35	Linalyl acetate	1249
35.024	0.04	Geranial	1266
40.324	0.1	Terpinyl acetate <alpha>	1345
41.088	2.07	Neryl acetate	1357
42.378	3.63	Geranyl acetate	1376
45.098	0.61	Caryophyllene <E>	1418
47.354	0.05	Humulene <alpha>	1454
49.839	0.18	Bicyclogermacrene	1494
51.217	0.02	Cadinene <delta>	1517
53.739	0.18	Nerolidol <E>	1559

24.) Bergamot

RT	Area%	Component	RI
12.22	0.34	Thujene <alpha>	922
12.637	1.38	Pinene <alpha>	930
13.558	0.03	Camphene	946
14.842	1.26	Sabinene	969
15.156	8.3	Pinene <beta>	975
15.781	0.78	Myrcene	986
16.671	0.02	Octanal	1001
16.862	0.02	Phellandrene <alpha>	1004
17.528	0.06	Terpienene <alpha>	1014
18.016	0.23	Cymene <para>	1022
18.356	37.79	Limonene	1027
18.442	0.04	Phellandrene <beta>	1028
18.717	0.04	Ocimene <Z,beta>	1032
19.409	0.07	Ocimene <E,beta>	1043
20.216	9.1	Terpinene <gamma>	1055
21.058	0.05	Mentha-3,8-diene <para>	1068
22.027	0.31	Terpinolene	1082
22.261	0.02	Fenchone	1086
22.997	8.5	Linalool	1097
23.358	0.03	n-Nonanal	1102
26.614	0.02	Citronellal	1149
28.592	0.03	Terpinen-4-ol	1177
29.598	0.08	Terpineol <alpha>	1192
30.387	0.01	Decanal	1203
30.672	0.03	Octyl acetate	1207
32.533	0.28	Neral	1234
33.365	29.48	Linalyl acetate	1246
34.557	0.51	Geranial	1264
39.843	0.03	Terpinyl acetate <alpha>	1343
40.045	0.01	Citronellyl acetate	1346
40.62	0.28	Neryl acetate	1354
41.91	0.2	Geranyl acetate	1374
44.178	0.02	Bergamotene <cis,alpha>	1409
44.587	0.11	Caryophyllene <trans>	1415
45.439	0.2	Bergamotene <alpha,trans>	1429
49.649	0.02	Bisabolene <Z,alpha>	1496
49.88	0.02	Farnesene <E,E,alpha>	1500
50.095	0.3	Bisabolene <beta>	1503

25.) Clementine

RT	Area%	Component	RI
11.299	0.01	Thujene <alpha>	923
11.686	0.64	Pinene <alpha>	930
13.793	0.75	Sabinene	971
14.084	0.07	Pinene <beta>	976
14.715	2.05	Myrcene	987
15.593	0.15	n-Octanal	1001
15.742	0.04	Phellandrene <alpha>	1003
15.874	0.09	Carene <delta-3>	1006
16.877	0.03	Cymene <para>	1021
17.244	94.89	Limonene	1027
18.238	0.03	Ocimene <E,beta>	1043
18.979	0.17	Terpinene <gamma>	1054
20.748	0.02	Terpinolene	1082
21.729	0.29	Linalool	1097
23.962	0.01	Limonene oxide <cis>	1129
24.249	0.02	Limonene oxide <trans>	1134
25.318	0.03	Citronellal	1149
28.245	0.05	Terpineol <alpha>	1192
29.036	0.3	n-Decanal	1204
31.563	0.03	Carvone	1241
33.702	0.01	Perilla aldehyde	1273
40.247	0.04	Copaene <alpha>	1373
41.044	0.03	Cubebene <beta>	1385
41.17	0.01	Elemene <beta>	1387
42.547	0.04	Dodecanal	1409
43.687	0.02	Copaene <beta>	1427
46.87	0.03	Germacrene D	1478
48.39	0.02	Farnesene <E,E,alpha>	1504
49.157	0.03	Cadinene <delta>	1517
62.301	0.1	Sinensal <alpha>	1749

26.) Shiikuwasha

RT	Area%	Component	RI
12.634	0.8	Thujene <alpha>	924
13.065	3.13	Pinene <alpha>	932
15.3	0.05	Sabinene	971
15.623	1.98	Pinene <beta>	977
16.247	1.86	Myrcene	988
17.12	0.06	Octanal	1003
17.352	0.09	Phellandrene <alpha>	1006
18.028	0.61	Terpienene <alpha>	1016
18.527	5.72	Cymene <para>	1024
18.891	55.35	Limonene	1029
18.974	0.05	Phellandrene <beta>	1030
19.922	0.29	Ocimene <E,beta>	1045
20.774	24.77	Terpinene <gamma>	1057
22.579	1.41	Terpinolene	1084
23.537	0.34	Linalool	1099
27.011	0.08	Terpineol <cis,beta>	1148
29.199	0.52	Terpinen-4-ol	1180
30.204	0.98	Terpineol <alpha>	1194
30.971	0.12	Decanal	1205
31.252	0.07	Octyl acetate	1209
40.482	0.05	Terpinyl acetate <alpha>	1345
42.429	0.08	Copaene <alpha>	1375
43.313	0.09	Elemene <beta>	1388
45.261	0.29	Caryophyllene <trans>	1419
47.522	0.08	Humulene <alpha>	1455
49.111	0.32	Germacrene D	1480
49.721	0.09	Viridiflorene	1490
50.009	0.23	Bicyclogermacrene	1494
51.372	0.18	Cadinene <delta>	1517
54.859	0.19	Spathulenol	1575
56.046	0.04	Cubeban-11-ol	1595
59.386	0.08	Cadinol <alpha>	1654

27.) Yuzu

RT	Area %	Component	RI
12.571	0.3	Thujene <alpha>	925
12.998	1.22	Pinene <alpha>	933
15.229	0.27	Sabinene	972
15.548	0.69	Pinene <beta>	978
16.171	1.72	Myrcene	989
17.037	0.23	n-Octanal	1003
18.443	4.29	Cymene <para>	1024
18.815	80.56	Limonene	1030
18.894	0.04	Phellandrene <beta>	1031
20.656	4.47	Terpinene <gamma>	1058
21.439	0.23	n-Octanol	1069
22.491	0.11	Terpinolene	1085
23.449	1.27	Linalool	1100
23.808	0.16	n-Nonanal	1105
25.761	0.21	Limonene oxide <cis>	1133
26.059	0.27	Limonene oxide <trans>	1137
28.423	0.27	n-Nonanol	1171
30.111	0.31	Terpineol <alpha>	1195
30.878	0.85	n-Decanal	1206
32.243	0.45	Citronellol	1226
33.042	0.06	Neral	1237
33.455	0.07	Carvone	1243
33.847	0.56	Geraniol	1249
35.07	0.1	Geranial	1267
36.559	0.1	Thymol	1289
40.262	0.33	Menthene <1,2-diol>	1344
44.228	0.4	Methyl anthranilate <methyl>	1404
44.508	0.26	Dodecanal	1409
49.714	0.08	Valencene	1492
67.183	0.12	Isovalencenol <E>	1799

28.) Lemon

RT	Area%	Component	RI
12.542	0.43	Thujene <alpha>	925
12.971	2.01	Pinene <alpha>	932
13.907	0.06	Camphene	949
15.203	1.59	Sabinene	972
15.54	12.47	Pinene <beta>	978
16.144	1.59	Myrcene	988
17.009	0.05	n-Octanal	1003
17.244	0.04	Phellandrene <alpha>	1007
17.918	0.21	Terpinene <alpha>	1017
18.419	0.72	Cymene <para>	1024
18.828	65.58	Limonene	1030
18.895	0.05	Phellandrene <beta>	1031
18.972	0.03	1,8-cineole	1032
19.113	0.05	Ocimene <Z,beta>	1034
19.811	0.1	Ocimene <E,beta>	1045
20.649	9.63	Terpinene <gamma>	1058
22.46	0.38	Terpinolene	1085
23.423	0.15	Linalool	1099
23.778	0.11	n-Nonanal	1104
27.069	0.09	Citronellal	1151
29.079	0.07	Terpinen-4-ol	1180
30.086	0.15	Terpineol <alpha>	1195
33.021	0.87	Neral	1237
35.054	1.54	Geranial	1267
41.133	0.53	Neryl acetate	1357
42.422	0.43	Geranyl acetate	1377
45.14	0.29	Caryophyllene <E>	1419
45.977	0.31	Bergamotene <alpha,trans>	1432
50.633	0.47	Bisabolene <beta>	1507

29.) Mandarin

RT	Area%	Component	RI
12.536	0.82	Thujene <alpha>	925
12.963	2.19	Pinene <alpha>	932
15.19	0.25	Sabinene	971
15.51	1.25	Pinene <beta>	977
16.132	1.65	Myrcene	988
16.999	0.15	n-Octanal	1003
17.231	0.05	Phellandrene <alpha>	1006
17.904	0.23	Terpinene <alpha>	1016
18.401	0.47	Cymene <para>	1024
18.773	70.07	Limonene	1029
18.852	0.17	Phellandrene <beta>	1031
20.636	20.16	Terpinene <gamma>	1057
22.449	0.75	Terpinolene	1085
23.407	0.13	Linalool	1099
23.521	0.06	Sabinene hydrate <trans>	1101
29.061	0.05	Terpinen-4-ol	1180
30.064	0.22	Terpineol <alpha>	1194
30.834	0.1	n-Decanal	1205
33.133	0.06	Ascaridole <Z>	1239
36.521	0.08	Thymol	1288
37.562	0.08	Ascaridole <E>	1303
44.177	0.55	Methyl anthranilate <methyl>	1404
45.109	0.11	Caryophyllene <E>	1418
50.373	0.13	Farnesene <E,E,alpha>	1502
64.361	0.22	Sinensal <alpha>	1747

30.) Myrrh

RT	Area%	Component	RI
19.765	0.21	Ocimene <E,beta>	1044
39.588	0.89	Elemene <delta>	1334
42.668	0.11	Elemene <beta,cis>	1381
42.788	0.31	Bourbonene <alpha>	1382
43.15	5.66	Elemene <beta,trans>	1388
45.074	0.74	Caryophyllene <E>	1418
45.686	2.17	Elemene <gamma>	1428
47.351	0.16	Humulene <alpha>	1454
48.938	1.16	Germacrene D	1479
49.437	0.42	Selinene <beta>	1487
49.736	32.48	Curzerene	1492
49.87	0.5	Selinene <alpha>	1494
50.136	0.15	Bisabolene <alpha>	1499
50.897	0.28	Amorphene <delta>	1511
51.199	0.14	Cadinene <delta>	1516
52.988	0.21	Elemol <alpha>	1546
53.644	2.38	Germacrene B	1557
54.654	0.54	Furanodiene	1574
55.924	0.12	Elemenone <trans,beta>	1596
57.526	29.11	Furanoeudesma-1,3-diene	1624
57.656	0.26	Spathulenol	1626
58.016	9.61	Lindestrene	1632
58.455	0.97	Cadinol <epi,alpha>	1640
59.505	0.25	Atractylone	1659
59.888	1.18	Intermedeol	1665
60.727	0.83	2-Methoxyfuranodiene A	1680
61.315	0.65	Germacrone	1691
62.376	7.43	2-Methoxyfuranodiene B	1710
71.132	0.88	Furanodiene <2-acetoxy>	1876
75.565	0.2	Furanoeudesmadiene	1966

31.) Cilantro (1)

RT	Area %	Component	RI
6.458	0.01	Methyl-alpha-methyl butyrate	931
12.411	0.03	Tricyclene	947
12.559	0.04	Thujene <alpha>	987
12.994	5.59	Pinene <alpha>	1026
13.924	0.89	Camphene	1027
15.216	0.22	Sabinene	1029
15.535	0.42	Pinene <beta>	1097
16.158	0.89	Myrcene	1166
17.932	0.04	Terpinene <alpha>	1169
18.422	0.99	Cymene <para>	1192
18.733	2.09	Limonene	1224
18.843	0.1	Phellandrene <beta>	1291
18.947	0.05	1,8-cineole	1365
20.649	3.64	Terpinene <gamma>	1373
21.474	0.18	Linalool oxide <cis>	1387
22.479	0.49	Terpinolene	1401
22.571	0.1	Linalool oxide <trans>	1427
23.642	73.89	Linalool	1452
23.754	0.08	Hotrienol	1457
26.806	4.52	Camphor	1480
27.093	0.04	Citronellal	1495
28.51	0.13	Borneol	1497
29.099	0.11	Terpinen-4-ol	1504
30.104	0.29	Terpineol <alpha>	1507
32.238	0.03	Citronellol	1510
33.86	1.37	Geraniol	1517
38.818	0.1	Myrtenyl acetate	1523
41.141	0.03	Neryl acetate	1526
42.441	3.59	Geranyl acetate	1560
45.152	0.05	Caryophyllene <E>	1614

32.) Cilantro (2)

RT	Area%	Component	RI
9.132	0.12	Hexenol <3Z>	850
9.529	0.18	Hexenol <2E>	860
11.152	0.83	n-Nonane	900
12.979	2.76	Pinene <alpha>	932
13.916	0.4	Camphene	949
15.526	0.15	Pinene <beta>	977
16.15	0.28	Myrcene	988
17.016	0.63	n-Octanal	1003
18.413	0.62	Cymene <para>	1024
18.719	0.79	Limonene	1029
20.633	3.86	Terpinene <gamma>	1057
22.47	0.16	Terpinolene	1085
23.476	31.55	Linalool	1100
26.767	1.97	Camphor	1147
30.24	0.41	Decenal <4E>	1197
30.868	9.88	n-Decanal	1206
33.729	0.38	Decenal <2Z>	1247
33.846	0.95	Geraniol	1249
34.729	20.31	Decenal <2E>	1262
35.121	10.79	Decen-1-ol <2E>	1268
35.382	2.83	n-Decanol	1271
37.817	0.49	Undecanal	1307
41.538	1.24	Undecenal <2E>	1364
41.84	0.22	Undecenol <2E>	1368
42.417	0.41	Geranyl acetate	1377
44.49	1.04	Dodecanal	1409
48.098	4.36	Dodecenal <2E>	1466
48.303	0.3	Dodecen-1-ol <2E>	1469
56.874	0.29	Tetradecanal	1612
60.212	1.8	Tetradeceneol <10E>	1671

33.) Cypress

RT	Area%	Component	RI
12.378	0.13	Tricyclene	922
12.526	0.67	Thujene <alpha>	924
12.979	46.7	Pinene <alpha>	932
13.786	0.65	Fenchene <alpha>	947
13.886	0.17	Camphene	948
15.178	1.87	Sabinene	971
15.494	0.86	Pinene <beta>	977
16.119	2.32	Myrcene	988
17.384	21.2	Carene <delta-3>	1009
17.889	0.42	Terpinene <alpha>	1016
18.375	0.66	Cymene <para>	1023
18.686	2.14	Limonene	1028
18.798	0.33	Phellandrene <beta>	1030
20.594	0.79	Terpinene <gamma>	1057
22.432	2.55	Terpinolene	1084
23.389	1.01	Linalool	1099
27.305	0.76	Karahanaenone	1155
29.043	1.95	Terpinen-4-ol	1180
30.045	0.68	Terpineol <alpha>	1194
36.861	0.62	Terpinen-4-ol acetate	1293
39.431	0.45	Terpinyl acetate	1332
40.316	5.58	Terpinyl acetate <alpha>	1345
44.836	1.01	Funebrene <beta>	1414
45.088	0.23	Caryophyllene <E>	1418
45.381	0.16	Cedrene <beta>	1423
47.347	0.25	Humulene <alpha>	1454
48.547	0.2	Cadina-1(6),4-diene <trans>	1473
48.936	1.5	Germacrene D	1479
51.195	0.56	Cadinene <delta>	1516
56.579	3.58	Cedrol	1607

34.) White Tumeric

RT	Area %	Component	RI
12.97	1.83	Pinene <alpha>	932
13.908	0.34	Camphene	949
15.199	0.25	Sabinene	972
15.521	2.93	Pinene <beta>	977
16.14	0.22	Myrcene	988
18.721	2	Limonene	1029
18.964	49.45	1,8-cineole	1032
23.422	3.16	Linalool	1099
28.481	0.24	Borneol	1172
29.072	0.75	Terpinen-4-ol	1180
29.813	1.54	Methyl salicylate	1191
30.079	1.61	Terpineol <alpha>	1194
45.122	0.7	Caryophyllene <E>	1419
47.188	2.13	Farnesene <E,beta>	1452
47.384	0.6	Humulene <alpha>	1455
47.656	0.27	Alloaromadendrene	1459
49.87	0.28	Cubebol <epi>	1494
50.045	0.49	Muurolene <alpha>	1497
50.932	0.87	Amorphene <delta>	1512
51.235	2.77	Cadinene <delta>	1517
53.029	6.06	Elemol	1547
53.772	0.79	Nerolidol <E>	1559
54.718	0.58	Germacrene-D-4-ol	1575
57.401	0.83	Eudesmol <10-epi,gamma>	1621
57.69	0.31	Cubenol <epi>	1627
57.924	1.81	Eudesmol <gamma>	1631
58.492	1.42	Muurolol <alpha>	1641
58.604	2.81	Murrolol <epi,alpha>	1643
58.754	0.7	Cadinol <delta>	1645
59.253	12.26	Cadinol <alpha>	1654

35.) Sotigrass

RT	Area %	Component	RI
6.255	0.1	Toluene	779
12.925	0.14	Pinene <alpha>	932
13.855	0.2	Camphene	949
15.045	1.39	3,7,7-trimethyl-1,3,5-Cycloheptatriene	970
16.08	0.62	Myrcene	988
16.195	0.25	1,8-cineole <dehydro>	990
16.705	48.29	Carene <delta-2>	1000
17.18	0.74	Phellandrene <alpha>	1006
17.855	0.27	Terpinene <alpha>	1016
18.34	0.37	Cymene <para>	1024
18.63	3.32	Limonene	1028
18.77	1.27	Phellandrene <beta>	1030
19.05	0.26	Ocimene <Z,beta>	1034
25.075	2.29	Menth-2-en-1-ol <cis,para>	1124
26.32	1.38	Menth-2-en-1ol <trans,para>	1142
28.355	0.99	Mentha-1,5-dien-8-ol <para>	1171
29.415	0.33	Cymen-8-ol <para>	1186
30.005	0.73	Terpineol <alpha>	1194
30.135	0.62	Piperitol <cis>	1196
30.965	0.6	Piperitol <trans>	1208
33.565	0.31	Car-3-en-2-one	1246
34.01	32.37	Piperitone	1254
41.06	0.17	Neryl acetate	1357
44.395	0.18	Decyl acetate	1408
45.04	1.01	Caryophyllene <E>	1419
45.755	0.18	Gurjunene <beta>	1430
49.405	0.54	Dihydroagarofuran	1488
51.16	0.37	Cadinene <delta>	1517
51.855	0.23	Kessane	1528
54.97	0.48	Caryophyllene oxide	1581

36.) Palmarosa

RT	Area%	Component	RI
15.83	0.04	Hept-5-en-2-one <6-methyl>	983
16.137	0.12	Myrcene	988
18.706	0.12	Limonene	1028
18.926	0.04	1,8-cineole	1032
19.103	0.28	Ocimene <Z,beta>	1034
19.803	1.15	Ocimene <E,beta>	1045
23.419	1.6	Linalool	1099
28.87	0.07	Menthol	1177
32.064	0.08	Nerol	1223
32.22	0.08	Citronellol	1225
33.009	0.55	Neral	1237
33.989	79.78	Geraniol	1251
35.051	1.17	Geranial	1267
37.11	0.32	Geranyl formate	1297
42.419	11.33	Geranyl acetate	1377
43.176	0.06	Elemene <beta>	1388
45.125	1.16	Caryophyllene <E>	1419
47.381	0.08	Humulene <alpha>	1455
51.179	0.06	Sesquiterpenoid	1516
53.459	0.12	Geranyl butanoate	1554
53.768	0.08	Nerolidol <E>	1559
55.045	0.29	Caryophyllene oxide	1581
59.173	0.09	Ageratochromene	1653
62.534	0.51	Farnesol <2E,6Z>	1713
64.389	0.6	Geranyl heptanoate	1747
68.782	0.05	Farnesyl acetate	1830
74.416	0.09	Geranyl octanoate	1942

37.) Citronella

RT	Area%	Component	RI
13.125	0.09	Pinene <alpha>	933
16.314	0.08	Myrcene	989
18.899	3.76	Limonene	1029
23.617	0.67	Linalool	1100
27.041	0.69	Isopulegol	1149
27.337	40.76	Citronellal	1153
31.051	0.11	n-Decanal	1206
32.27	0.1	Nerol	1224
32.45	11.16	Citronellol	1227
33.22	0.36	Neral	1238
34.112	26.28	Geraniol	1251
35.247	0.55	Geranial	1267
40.029	0.1	Citridiol A	1339
40.744	1.93	Citronellyl acetate	1349
40.825	0.47	Eugenol	1351
42.616	2.28	Geranyl acetate	1378
43.038	0.1	Bourbonene <alpha>	1384
43.394	1.39	Elemene <beta>	1390
45.342	0.24	Caryophyllene <trans>	1420
48.798	0.1	Cadina-1(6),4-diene <trans>	1475
49.194	1.88	Germacrene D	1481
50.259	0.31	Muurolene <alpha>	1498
51.149	0.76	Amorphene <delta>	1513
51.449	1.32	Cadinene <delta>	1518
53.244	2.31	Elemol <alpha>	1548
54.939	1.16	Germacrene D-4-ol	1577
58.707	0.16	Muurolol <alpha>	1642
58.815	0.2	Murrolol <epi,alpha>	1644
59.463	0.68	Cadinol <alpha>	1655

38.) Cardamom

RT	Area%	Component	RI
12.539	0.24	Thujene <alpha>	925
12.967	1.7	Pinene <alpha>	932
15.198	4.52	Sabinene	972
15.515	0.39	Pinene <beta>	977
16.138	2.16	Myrcene	988
17.005	0.11	n-Octanal	1003
17.91	0.08	Terpinene <alpha>	1016
18.401	0.26	Cymene <para>	1024
18.714	2.54	Limonene	1029
18.942	35.46	1,8-cineole	1032
20.618	0.2	Terpinene <gamma>	1057
21.43	0.57	Sabinene hydrate <cis>	1069
22.457	0.07	Terpinolene	1085
23.421	3.65	Linalool	1099
23.532	0.27	Sabinene hydrate <trans>	1101
24.359	0.06	2-Methyl-6-methylen-octa-1,7-dien-3-one	1113
28.347	0.08	Terpineol <delta>	1170
29.075	0.74	Terpinen-4-ol	1180
30.081	1.75	Terpineol <alpha>	1194
31.129	0.09	Octyl acetate	1210
31.55	0.38	Sabinene hydrate acetate <cis>	1216
33.011	0.16	Neral	1237
33.82	5.51	Linalyl acetate	1249
35.041	0.25	Geranial	1267
38.148	0.12	Terpinyl acetate <delta>	1312
38.665	0.07	Methyl geranate	1320
40.387	37.29	Terpinyl acetate <alpha>	1346
42.411	0.62	Geranyl acetate	1377
49.479	0.09	Selinene <beta>	1488
53.777	0.57	Nerolidol <E>	1560

39.) Pitanga

RT	Area%	Component	RI
40.532	0.74	Cubebene <alpha>	1346
42.437	8.17	Copaene <alpha>	1375
43.318	2.59	Elemene <beta>	1388
45.279	13.97	Caryophyllene <trans>	1419
45.855	1.06	Elemene <gamma>	1428
47.523	2.18	Humulene <alpha>	1455
47.794	2.65	Alloaromadendrene	1459
48.545	1.33	Cadina-1(6),4-diene <cis>	1471
48.726	3.85	Cadina-1(6),4-diene <trans>	1474
49.115	2.16	Germacrene D	1480
49.448	1.71	Guaiene <cis,beta>	1485
49.729	3.06	Viridiflorene	1490
50.014	2.32	Bicyclogermacrene	1494
50.187	3.97	Muurolene <alpha>	1497
50.63	0.59	Guaiene <trans,beta>	1504
51.396	14.22	Cadinene <delta>	1517
51.587	4.18	Calamenene <trans>	1520
51.669	1.65	Zonarene	1522
52.231	0.64	Cadine-1,4-diene <trans>	1531
53.821	1.49	Germacrene B	1558
54.871	3.05	Spathulenol	1576
55.196	2.07	Caryophyllene oxide	1581
55.419	4.02	Globulol	1585
55.922	1.96	Viridiflorol	1593
56.053	1.44	Cubeban-11-ol	1595
56.656	1.05	Rosifoliol	1606
57.845	4.96	Cubenol <epi>	1627
58.707	5.24	Cubenol	1642
58.903	1.35	Cadinol <delta>	1645
59.395	2.33	Cadinol <alpha>	1654

40.) Galbanum Resin

RT	Area %	Component	RI
11.315	0.67	Thujene <alpha>	923
11.71	5.96	Pinene <alpha>	931
12.58	0.13	Camphene	948
13.825	0.78	Sabinene	971
14.185	77.18	Pinene <beta>	978
14.736	3.21	Myrcene	988
15.893	1.48	3-Carene	1006
16.88	0.65	Cymene <para>	1021
17.164	1.7	Limonene	1026
17.264	0.48	Phellandrene <beta>	1027
17.575	0.53	Ocimene <Z,beta>	1032
24.503	0.59	Pinocarveol <trans>	1137
25.982	0.19	Pinocarvone	1159
28.27	0.8	Myrtenal	1193
29.765	0.47	Fenchyl acetate <endo>	1215
31.201	0.55	Thymol, methyl ether	1236
31.637	0.63	3-methyl-2-butenyl tiglate	1242
34.288	0.15	Bornyl acetate	1282
38.409	0.74	Terpinyl acetate <alpha>	1345
39.852	0.14	Ylangene <alpha>	1367
40.273	0.25	Copaene <alpha>	1373
45.138	0.13	Brasila-1(6),5(10)-diene	1451
47.01	0.3	Himachalene <gamma>	1481
48.375	0.27	Dihydroagarofuran <beta>	1503
51.718	0.27	Muurol-5-en-4-a-ol <Z>	1560
52.957	0.26	Juneol <10-epi>	1581
53.81	0.45	Guaiol	1596
55.26	0.25	Eudesmol <10-epi,gamma>	1621
57.685	0.79	Bulnesol	1664

41.) Fennel

RT	Area %	Component	RI
6.295	0.01	Cyclohexene <3-methylene>	779
12.415	0.02	Tricyclene	922
12.562	0.02	Thujene <alpha>	925
12.993	4.44	Pinene <alpha>	932
13.826	0.04	Fenchene <alpha>	947
13.928	0.46	Camphene	949
15.221	0.23	Sabinene	971
15.539	0.23	Pinene <beta>	977
16.164	1.71	Myrcene	988
17.264	0.57	Phellandrene <alpha>	1006
17.937	0.02	Terpinene <alpha>	1016
18.425	0.14	Cymene <para>	1024
18.737	2.99	Limonene	1028
18.848	0.6	Phellandrene <beta>	1030
18.954	0.15	1,8-cineole	1032
19.126	0.05	Ocimene <Z,beta>	1034
20.646	1.07	Terpinene <gamma>	1057
21.465	0.16	Sabinene hydrate <cis>	1069
22.491	0.23	Terpinolene	1085
22.785	25.53	Fenchone	1089
24.831	0.03	Fenchol <endo>	1119
25.313	0.08	Pinene hydrate <trans>	1126
26.787	0.54	Camphor	1147
29.106	0.02	Terpinen-4-ol	1180
30.307	3.3	Methyl chavicol	1197
34.05	0.11	Anethole <Z>	1252
34.149	0.11	Anis aldehyde <para>	1253
36.476	57.06	Anethole <E>	1287
42.478	0.05	2-Methoxyphenylacetone	1377
49.007	0.03	Germacrene D	1480

42.) Wintergreen

RT	Area%	Component	RI
12.966	0.05	Pinene <alpha>	932
13.905	0.01	Camphene	948
15.291	0	Phenol	973
15.515	0.01	Pinene <beta>	977
17.232	0	Phellandrene <alpha>	1006
18.399	0.01	Cymene <ortho>	1023
18.704	0	Limonene	1028
18.933	0.01	Benzyl alcohol	1031
21.45	0.01	Octanol	1069
23.42	0.05	Linalool	1099
28.315	0.01	Ethyl benzoate	1169
30.112	99.65	Methyl salicylate	1194
33.842	0.02	Geraniol	1249
35.066	0.16	Ethyl salicylate	1266
45.138	0.01	Caryophyllene <E>	1419

43.) Zdravetz

RT	Area%	Component	RI
17.896	0.37	Terpinene <alpha>	1016
18.386	0.91	Cymene <para>	1024
18.693	0.29	Limonene	1028
19.788	0.55	Ocimene <E,beta>	1045
20.605	3.43	Terpinene <gamma>	1057
22.442	0.57	Terpinolene	1084
23.404	0.79	Linalool	1099
29.062	0.44	Terpinen-4-ol	1180
43.179	0.97	Elemene <beta>	1388
45.72	5.55	Elemene <gamma>	1428
49.471	0.35	Selinene <beta>	1488
50.486	0.98	Farnesene <E,E,alpha>	1504
52.096	0.53	Zonarene	1531
52.371	1.35	Eremophilene	1536
52.643	1.45	Selina-3,7(11)-diene	1541
52.826	0.76	Occidentalol	1544
53.685	10.06	Germacrene B	1558
55.966	8.01	Elemenone <cis,beta>	1596
57.51	0.49	Eremoligenol	1623
57.927	1.51	Eudesmol <gamma>	1631
59.265	6.87	Eudesmol <alpha>	1654
59.742	2.1	Germacrone A	1663
60.944	3.02	Bisabolol <epi,alpha>	1684
61.403	40.08	Germacrone	1692
61.645	1.06	Eudesm-7(11)-en-4-ol	1696
63.337	4.05	Germacrone B	1728

44.) Ginger Lily (1)

RT	Area %	Component	RI
21.733	6.2	Linalool	1097
25.971	3.94	Benzyl acetate	1159
31.986	1.84	Geraniol	1248
37.921	2.88	Anthanilate methyl	1337
39.286	1.21	Jasmine Sesquiterpene A	1358
40.835	1.94	Methyl cinnamate <E>	1382
41.355	5.56	Jasmone <cis>	1390
42.435	1.13	Jasmine sesquiterpene B	1407
43.038	2.08	Caryophyllene <trans>	1417
43.379	1	Gurjunene <beta>	1422
44.123	2.06	Guaiene <alpha>	1434
44.554	0.91	p-Methoxypropiophenone	1441
44.788	1.17	Amorpha-4,11-diene	1445
44.965	7.12	Clovene <alpha>	1448
45.511	1.1	Rotundene	1456
46.331	1.86	Gurjunene <gamma>	1470
46.734	1.88	Curcumene <gamma>	1476
47.484	1.77	Alaskene <beta>	1488
48.15	4.1	Bisabolene <alpha>	1500
48.579	2.71	Bisabolene <beta>	1507
48.732	8.45	2,6,10,10-Tetramethylbicyclo[7.2.0]undeca-2,6-diene	1509
49.69	3.65	Bisabolene <E,gamma>	1526
49.924	1.39	Nerolidol <cis>	1530
50.55	16.49	Bisabolene <trans,alpha>	1540
51.76	2.61	Nerolidol <E>	1560
52.35	10.92	Hexenyl Benzoate <3Z>	1570
63.193	2.45	Benzyl benzoate	1766

45.) Ginger Lily (2)

RT	Area %	Component	RI
23.431	13.02	Linalool	1099
27.745	7.23	Benzyl acetate	1161
33.835	3.11	Geraniol	1249
39.868	4.94	Anthranilate <methyl>	1338
40.626	2.07	Eugenol	1350
41.288	1.09	Jasmine Sesquiterpene A	1360
42.786	2.9	Methyl cinnamate <E>	1382
43.33	8.37	Jasmone <Z>	1391
45.129	3.17	Caryophyllene <trans>	1419
45.993	0.89	Jasmine Sesquiterpene C	1433
46.954	1.36	Clovene <alpha>	1448
48.768	2.04	Curcumene <gamma>	1477
48.959	0.99	Curcumene	1480
49.259	0.83	Himachalene <gamma>	1485
50.169	2.19	Bisabolene <Z,alpha>	1499
50.616	2.06	Bisabolene <beta>	1506
50.771	5.4	2,6,10,10-Tetramethylbicyclo[7.2.0]undeca-2,6-diene	1509
51.751	2.12	Bisabolene <E,gamma>	1526
51.932	2.36	Nerolidol <cis>	1529
52.571	10.16	Bisabolene <trans,alpha>	1539
53.782	4.34	Nerolidol <E>	1560
54.381	12.82	Hexenyl Benzoate <3Z>	1570
58.83	2.31	Methyl dihydrojasmonate <cis>	1647
65.31	1.82	Benzyl benzoate	1764

46.) Helichrysum

RT	Area %	Component	RI
8.483	0.15	Hexan-3-one <4-methyl>	834
12.974	26.85	Pinene <alpha>	932
13.788	0.19	Fenchene <alpha>	947
15.498	0.22	Pinene <beta>	977
18.689	2.17	Limonene	1028
18.901	0.17	1,8-cineole	1031
19.987	0.38	Isobutyl angelate	1048
20.596	0.21	Terpinene <gamma>	1057
23.391	0.42	Linalool	1099
26.949	1.35	Isoamyl tiglate	1150
29.062	0.71	2,2,5,5-tetramethyl-3,4-hexanedione	1180
30.048	0.22	Terpineol <alpha>	1194
32.028	0.33	Nerol	1223
36.091	0.54	Hexyl angelate	1282
41.088	5.01	Neryl acetate	1357
41.822	0.44	Ylangene <alpha>	1368
42.271	1.45	Copaene <alpha>	1375
44.198	6.52	Italicene	1404
44.672	0.83	Bergamotene <alpha,cis>	1411
45.097	5.48	Caryophyllene <E>	1418
46.834	0.59	Neryl propanoate	1446
47.868	0.46	Acoradiene <beta>	1462
48.757	27.62	Curcumene <gamma>	1477
48.936	4.07	Curcumene	1479
49.137	0.5	Neryl isobutanoate	1483
49.456	7.49	Selinene <beta>	1488
49.885	3.63	Selinene <alpha>	1495
50.015	0.22	Muurolene <alpha>	1497
50.682	0.72	Curcumene <beta>	1508
50.907	0.43	Amorphene <delta>	1511
51.202	0.63	Cadinene <delta>	1516

47.) Ghandi Root

RT	Area %	Component	RI
12.574	0.41	Thujene <alpha>	925
15.233	1.74	Sabinene	972
16.172	0.43	Myrcene	989
17.425	1.45	3-Carene <delta>	1009
17.949	1.39	Terpinene <alpha>	1017
18.439	2.17	Cymene <para>	1024
18.746	1.01	Limonene	1029
18.858	0.39	Phellandrene <beta>	1031
20.659	2.62	Terpinene <gamma>	1058
21.491	0.49	Linalool oxide <cis>	1070
22.498	0.7	Terpinolene	1085
22.59	0.35	Linalool oxide <trans>	1087
23.546	60.13	Linalool	1101
23.714	0.61	Hotrienol	1103
29.131	11.18	Terpinen-4-ol	1181
30.119	2	Terpineol <alpha>	1195
32.097	0.45	Nerol	1224
33.867	0.55	Geraniol	1249
47.424	0.28	Humulene <alpha>	1455
49.942	0.51	Selinene <alpha>	1495
50.082	0.28	Muurolene <alpha>	1498
50.971	0.47	Amorphene <delta>	1512
51.272	2	Cadinene <delta>	1517
54.757	1.42	Spathulenol	1576
57.153	0.4	Selina-6-en-4-ol	1617
58.52	0.33	Muurolol <alpha>	1641
58.636	1.12	Murrolol <epi,alpha>	1643
59.285	2.65	Cadinol <alpha>	1655
61.324	2.13	Sesquiterpene alchol	1691
62.756	0.34	Eudesma-4,11-dien-2-ol	1717

48.) Hyssop

RT	Area %	Component	RI
11.295	0.22	Thujene <alpha>	923
11.683	0.76	Pinene <alpha>	930
13.791	1.85	Sabinene	971
14.09	10.75	Pinene <beta>	976
14.71	1.82	Myrcene	987
16.858	0.12	Cymene <para>	1021
17.144	3.62	Limonene	1026
17.245	3.75	Phellandrene <beta>	1027
17.33	0.33	1,8-cineole	1028
18.21	0.56	Ocimene <E,beta>	1042
21.729	0.7	Linalool	1097
22.179	0.2	Thujone <alpha>	1103
22.943	0.13	Thujone <beta>	1115
25.861	13.18	Pinocamphone <trans>	1157
25.975	0.2	Pinocarvone	1159
27.006	45.87	Pinocamphone <cis>	1174
27.227	0.26	Terpinen-4-ol	1177
28.259	2.22	Myrtenol	1193
36.835	0.4	Myrtenyl acetate <cis>	1320
40.759	1.09	Bourbonene <alpha>	1380
42.254	0.31	Gurjunene <alpha>	1404
43.035	0.87	Caryophyllene <trans>	1417
45.546	1.36	(-)-Alloaromadendrene	1457
46.875	2.45	Germacrene D	1478
47.763	2.04	Bicyclogermacrene	1493
48.841	0.23	Amorphene <delta>	1511
50.949	1.62	Elemol <alpha>	1547
52.589	0.96	Spathulenol	1574
55.801	0.24	Eudesmol <gamma>	1631

49.) Star Anise

RT	Area%	Component	RI
12.927	0.47	Pinene <alpha>	931
15.151	0.03	Sabinene	971
15.469	0.04	Pinene <beta>	976
16.091	0.08	Myrcene	987
17.19	0.25	Phellandrene <alpha>	1006
17.337	0.24	3-Carene <delta>	1008
17.861	0.06	Terpinene <alpha>	1016
18.351	0.07	Cymene <para>	1023
18.658	1.39	Limonene	1028
18.769	0.27	Phellandrene <beta>	1029
18.867	0.21	1,8-cineole	1031
20.565	0.07	Terpinene <gamma>	1056
22.403	0.05	Terpinolene	1084
23.358	0.9	Linalool	1098
29.007	0.14	Terpinen-4-ol	1179
30.012	0.12	Terpineol <alpha>	1193
30.212	3.01	Methyl chavicol	1196
33.956	0.19	Anethole <Z>	1251
34.056	0.3	Anis aldehyde <para>	1252
36.395	90.7	Anethole <E>	1286
42.225	0.05	Copaene <alpha>	1374
42.541	0.07	Anisyl methyl ketone	1379
44.636	0.04	Bergamotene <alpha,cis>	1411
45.054	0.22	Caryophyllene <E>	1418
45.888	0.25	Bergamotene <alpha,trans>	1431
50.308	0.02	Farnesene <E,E,alpha>	1501
50.536	0.04	Bisabolene <beta>	1505
53.696	0.03	Nerolidol <E>	1558
54.23	0.04	Anethole hydrate	1567
60.362	0.65	Foeniculin	1674

50.) Juniper

RT	Area%	Component	RI
12.539	2.25	Thujene <alpha>	925
13.01	33.35	Pinene <alpha>	933
13.897	0.21	Camphene	949
15.218	16.39	Sabinene	972
15.515	2.43	Pinene <beta>	977
16.161	14.62	Myrcene	989
17.231	0.15	Phellandrene <alpha>	1006
17.904	1.3	Terpinene <alpha>	1016
18.395	1.43	Cymene <para>	1024
18.713	5.72	Limonene	1029
18.818	0.65	Phellandrene <beta>	1030
20.614	2.53	Terpinene <gamma>	1057
22.45	1.52	Terpinolene	1085
29.076	3.99	Terpinen-4-ol	1180
30.066	0.26	Terpineol <alpha>	1194
40.388	0.4	Cubebene <alpha>	1346
42.282	0.18	Copaene <alpha>	1375
43.169	1.16	Elemene <beta>	1388
45.113	1.4	Caryophyllene <E>	1419
45.708	0.75	Elemene <gamma>	1428
47.178	0.32	Farnesene <E,beta>	1451
47.371	1.14	Humulene <alpha>	1454
48.574	0.27	Cadina-1(6),4-diene <trans>	1474
48.969	3.9	Germacrene D	1480
49.87	0.38	Bicyclogermacrene	1494
50.032	0.27	Muurolene <alpha>	1497
50.918	0.41	Amorphene <delta>	1512
51.222	1.07	Cadinene <delta>	1517
53.667	1.36	Germacrene B	1558
54.702	0.19	Spathulenol	1575

51.) White Kunzea

RT	Area%	Component	RI
6.954	0.17	2,4-dimethyl-3-pentanone	796
12.613	0.15	Thujene <alpha>	926
13.079	41.62	Pinene <alpha>	934
15.274	0.47	Sabinene	973
15.594	0.66	Pinene <beta>	979
16.215	0.26	Myrcene	989
17.993	0.15	Terpinene <alpha>	1018
18.485	0.47	Cymene <para>	1025
18.796	1.06	Limonene	1030
19.027	16.94	1,8-cineole	1033
19.885	0.41	Ocimene <E,beta>	1046
20.702	0.31	Terpinene <gamma>	1058
23.508	1.05	Linalool	1100
23.97	0.3	Isopentyl isovalerate	1107
25.404	0.2	Campholenal <alpha>	1128
29.166	0.54	Terpinen-4-ol	1181
30.173	1.79	Terpineol <alpha>	1196
32.304	1.11	Citronellol	1227
44.431	0.39	Gurjunene <alpha>	1408
45.22	0.47	Caryophyllene <E>	1420
47.753	0.88	Alloaromadendrene	1461
49.684	2.54	Viridiflorene	1491
49.97	3.53	Bicyclogermacrene	1496
51.328	0.66	Cadinene <delta>	1518
51.528	1.17	Calamenene <trans>	1522
54.455	0.47	Palustrol	1571
54.819	0.39	Spathulenol	1577
55.368	2.47	Globulol	1586
55.909	17.31	Viridiflorol	1595
56.475	2.06	Ledol	1605

52.) Kanuka

RT	Area%	Component	RI
6.901	0.3	2,4-dimethyl-3-pentanone	794
10.007	0.2	Isopentyl acetate	872
12.537	1.64	Thujene <alpha>	925
13.006	66.28	Pinene <alpha>	933
15.507	0.62	Pinene <beta>	977
17.902	0.14	Terpinene <alpha>	1016
18.397	5.49	Cymene <para>	1024
18.7	1.45	Limonene	1028
18.916	2.59	1,8-cineole	1032
19.793	0.42	Ocimene <E,beta>	1045
20.446	0.25	Pentyl isobutanoate	1054
20.617	7.13	Terpinene <gamma>	1057
22.449	1.39	Terpinolene	1085
23.409	1.88	Linalool	1099
23.873	0.3	Isopentyl isovalerate	1106
25.308	0.28	Campholenal <alpha>	1126
26.291	0.23	Pinocarveol <trans>	1140
29.062	0.27	Terpinen-4-ol	1180
30.067	0.56	Terpineol <alpha>	1194
47.641	0.2	Alloaromadendrene	1459
49.564	0.27	Viridiflorene	1489
49.856	0.5	Bicyclogermacrene	1494
51.219	0.25	Cadinene <delta>	1517
51.417	0.79	Calamenene <trans>	1520
52.076	0.33	Cadina-1,4-diene <trans>	1531
53.765	1.71	Nerolidol <E>	1559
54.347	0.23	Palustrol	1569
54.707	0.56	Spathulenol	1575
55.763	2.91	Viridiflorol	1593
56.36	0.83	Ledol	1603

53.) Laurel Leaf

RT	Area%	Component	RI
12.526	0.59	Thujene <alpha>	924
12.956	5.89	Pinene <alpha>	932
13.889	0.53	Camphene	948
15.187	7.16	Sabinene	971
15.503	4.41	Pinene <beta>	977
16.121	0.75	Myrcene	988
17.221	0.2	Phellandrene <alpha>	1006
17.37	0.21	Carene <delta-3>	1008
17.893	0.69	Terpinene <alpha>	1016
18.386	0.62	Cymene <para>	1024
18.703	1.62	Limonene	1028
18.82	0.59	Phellandrene <beta>	1030
18.954	47.72	1,8-cineole	1032
19.783	0.23	Ocimene <E,beta>	1045
20.6	1.24	Terpinene <gamma>	1057
21.411	0.13	Sabinene hydrate <cis>	1069
22.436	0.31	Terpinolene	1084
23.408	4.79	Linalool	1099
28.323	0.23	Terpineol <delta>	1169
28.457	0.21	Borneol	1171
29.054	2.93	Terpinen-4-ol	1180
30.055	1.95	Terpineol <alpha>	1194
36.187	0.3	Bornyl acetate	1283
38.118	0.52	Terpinyl acetate <delta>	1312
40.339	10.51	Terpinyl acetate <alpha>	1345
40.602	0.77	Eugenol	1349
43.154	0.29	Elemene <beta>	1388
43.781	3.88	Methyleugenol	1397
45.099	0.58	Caryophyllene <trans>	1418
55.019	0.15	Caryophyllene oxide	1580

54.) Lavandin

RT	Area%	Component	RI
12.913	0.38	Pinene <alpha>	931
13.847	0.25	Camphene	948
15.46	0.47	Octen-3-ol	976
16.074	0.44	Myrcene	987
17.468	0.15	Hexyl acetate	1010
18.64	0.65	Limonene	1027
18.852	5.35	1,8-cineole	1031
19.032	0.91	Ocimene <Z,beta>	1033
19.733	0.24	Ocimene <E,beta>	1044
21.359	0.34	Sabinene hydrate <cis>	1068
22.381	0.16	Terpinolene	1083
23.393	34.4	Linalool	1099
23.861	0.27	Octen-3-yl acetate	1105
26.682	7.42	Camphor	1146
27.775	0.55	Lavandulol	1161
28.406	2.99	Borneol	1170
28.998	3.79	Terpinen-4-ol	1179
29.749	0.46	Hexyl butanoate	1190
29.998	0.73	Terpineol <alpha>	1193
33.233	0.17	Hexyl isovalerate	1240
33.773	33.09	Linalyl acetate	1248
36.047	2.56	Lavandulyl acetate	1281
39.135	0.16	Hexyl tiglate	1327
42.331	0.18	Geranyl acetate	1375
45.048	1.65	Caryophyllene <E>	1418
47.113	1.25	Farnesene <E,beta>	1450
48.898	0.59	Germacrene D	1479
50.164	0.25	Lavandulyl isovalerate	1499
60.853	0.15	Bisabolol <alpha>	1682

55.) May Chang

RT	Area %	Component	RI
11.826	1.39	Pinene <alpha>	933
12.706	0.34	Camphene	950
13.946	0.58	Sabinene	974
14.238	0.98	Pinene <beta>	979
14.627	1.12	Hept-5-en-2-one <6-methyl>	986
14.871	1.31	Myrcene	990
17.316	13.31	Limonene	1028
17.505	1.56	1,8-cineole	1031
21.906	1.19	Linalool	1099
25.485	1.51	Citronellal	1152
26.115	0.56	Chrysanthenol <cis>	1161
26.814	0.16	Borneol	1171
27.41	1.39	Isocitral <E>	1180
28.428	0.61	Terpineol <alpha>	1195
30.431	0.29	Nerol	1225
30.615	0.21	Citronellol	1227
31.373	30.55	Neral	1239
32.195	0.75	Geraniol	1251
33.404	40.23	Geranial	1269
41.354	0.19	Elemene <beta>	1390
43.236	1.46	Caryophyllene <trans>	1420
45.481	0.19	Humulene <alpha>	1456
53.08	0.12	Caryophyllene oxide	1583

56.) Blue Chamomile

RT	Area%	Component	RI
12.956	0.08	Pinene <alpha>	932
18.388	0.15	Cymene <para>	1024
19.089	0.13	Ocimene <Z,beta>	1034
19.789	0.85	Ocimene <E,beta>	1045
20.55	0.41	Artemisia ketone	1056
20.605	0.19	Terpinene <gamma>	1057
39.39	0.12	Elemene <delta>	1331
43.112	0.63	Isocomene <alpha>	1387
45.105	0.51	Caryophyllene <E>	1418
47.216	60.22	Farnesene <E,beta>	1452
47.708	0.18	Ageratochromene <6-demethoxy>	1460
48.964	3	Germacrene D	1480
49.529	1.07	Farnesene	1489
49.853	2.42	Bicyclogermacrene	1494
50.376	8.44	Farnesene <E,E,alpha>	1502
50.598	0.16	Bisabolene <beta>	1506
51.214	0.12	Cadinene <delta>	1517
53.739	0.17	Nerolidol <E>	1559
54.234	0.17	Dendrolasin	1567
54.698	0.43	Spathulenol	1575
57.555	0.11	Bisabolendienol	1624
59.171	2.78	Bisabolol oxide B <alpha>	1653
60.669	1.95	Bisabolone oxide A <alpha>	1679
60.908	0.71	Bisabolol <alpha>	1683
63.277	0.42	Chamazulene	1726
64.343	11.42	Bisabolol oxide A <alpha>	1746
69.241	0.21	Phytone	1839
71.11	2.58	Spiroether <Z>	1876
71.826	0.19	Spiroether <E>	1890

57.) Cajeput

RT	Area %	Component	RI
6.85	0.06	2,4-dimethyl-3-pentanone	794
12.445	0.26	Thujene <alpha>	924
12.85	4.15	Pinene <alpha>	932
15.4	1.49	Pinene <beta>	976
16.025	0.59	Myrcene	987
17.145	0.18	Phellandrene <alpha>	1006
17.285	0.17	Carene <delta-3>	1008
17.805	0.25	Terpinene <alpha>	1016
18.28	1.53	Cymene <para>	1023
18.58	6.31	Limonene	1028
18.78	66.31	1,8-cineole	1032
20.49	2.12	Terpinene <gamma>	1056
22.335	0.76	Terpinolene	1084
23.3	0.3	Linalool	1098
28.24	0.1	Terpineol <delta>	1169
28.945	0.54	Terpinen-4-ol	1179
29.93	6.57	Terpineol <alpha>	1194
40.205	1.05	Terpinyl acetate <alpha>	1345
41.75	0.17	Ylangene <alpha>	1368
43.065	0.15	Elemene <beta,trans>	1388
44.965	2.79	Caryophyllene <E>	1418
47.235	1.33	Humulene <alpha>	1454
48.37	0.12	Gurjunene <gamma>	1471
48.58	0.26	Himachalene <gamma>	1475
48.73	0.29	Amorphene <alpha>	1477
49.225	0.19	Selinene <delta>	1485
49.345	0.94	Selinene <beta>	1487
49.755	0.8	Aciphyllene	1494
54.93	0.1	Caryophyllene oxide	1580
55.65	0.12	Viridiflorol	1592

58.) Myrtle

RT	Area %	Component	RI
6.924	0.15	2,4-dimethyl-3-pentanone	795
11.856	0.93	Isobutyl isobutyrate	913
12.566	0.3	Thujene <alpha>	925
13.01	22.47	Pinene <alpha>	933
15.542	0.28	Pinene <beta>	978
16.165	0.28	Myrcene	989
16.242	0.19	Linalool oxide <dehydroxy,trans>	990
17.176	0.12	Linalool oxide <dehydroxy,cis>	1005
17.264	0.14	Phellandrene <alpha>	1007
17.415	0.49	Carene <delta-3>	1009
18.432	1.51	Cymene <para>	1024
18.756	12.14	Limonene	1029
18.988	35	1,8-cineole	1033
19.829	0.36	Ocimene <E,beta>	1045
20.647	0.42	Terpinene <gamma>	1057
22.484	0.32	Terpinolene	1085
23.464	7.92	Linalool	1100
23.68	0.13	Hotrienol	1103
29.102	0.23	Terpinen-4-ol	1180
30.113	2.59	Terpineol <alpha>	1195
30.296	0.27	Methyl chavicol	1198
33.846	1.18	Linalyl acetate	1249
38.836	7.92	Myrtenyl acetate	1323
40.378	0.64	Terpinyl acetate <alpha>	1346
41.146	0.09	Neryl acetate	1358
42.44	2.28	Geranyl acetate	1377
43.827	0.45	Methyleugenol	1398
45.156	0.41	Caryophyllene <E>	1419
47.417	0.69	Humulene <alpha>	1455
50.977	0.1	Dihydoquinone	1513

59.) Spikenard

RT	Area%	Component	RI
12.931	2.88	Pinene <alpha>	932
15.476	5.12	Pinene <beta>	976
36.896	1.04	Methyl myrtenate	1293
42.84	3.78	Patchoulene <beta>	1383
44.547	1.33	Gurjunene <alpha>	1409
45.011	1.02	Aristolene	1417
45.546	0.84	Aromadendrene dehydro	1425
45.773	9.94	Gurjunene <beta>	1429
46.057	1.79	Guaiene <alpha>	1434
46.136	1.35	Vatirenene <beta>	1435
46.448	12.92	6,9-guaiadiene	1440
46.944	4.7	Seychellene	1448
47.148	8.03	Velerana-7,11-diene	1451
47.6	0.86	Alloaromadendrene	1458
47.709	2.78	Patchoulene <alpha>	1460
47.903	2.18	Caryophyllene <9-epi>	1463
49.13	2.09	Muurola-4(14),5-diene <trans>	1483
49.422	1.07	Selinene <beta>	1487
49.635	4.62	Valencene	1491
49.885	1.48	Guaine <E,beta>	1495
51.296	10.44	Nordosina7,9,11-triene <E>	1518
55.006	3.2	Nardol	1580
56.214	1.68	Carotol	1600
59.659	1.95	1(10)-Aristolen-9-beta-ol	1661
60.098	1.1	Patchouli alcohol	1669
60.262	4.81	Jatamansone	1672
65.631	0.74	9-Hydroxyaristol-1(10)-en-2-one	1770

60.) Blue Lotus

RT	Area %	Component	RI
18.715	0.13	Limonene	1029
18.936	0.69	1,8-cineole	1032
23.047	0.23	Methyl benzoate	1093
28.63	16.72	Methyl benzeneacetate	1174
40.628	0.14	Eugenol	1350
41.53	0.36	Clovene <alpha>	1363
41.866	0.12	Ylangene <alpha>	1368
43.395	0.63	Blue Lotus	1392
44.175	1.28	Caryophyllene <cis>	1404
44.574	0.73	Isocaryophyllene	1410
44.87	0.39	Caryophyllene <trans>	1415
45.136	65.56	Caryophyllene <gamma>	1419
45.654	2.22	Ylangene <beta>	1427
45.759	0.65	Elemene <gamma>	1429
46.996	0.87	Spirolepechinene	1449
47.387	0.97	Humulene <alpha>	1455
55.05	1.87	Caryophyllene oxide	1581
60.152	0.38	Patchouli alcohol	1670
76.915	0.57	Olealdehyde	1993
83.012	0.26	Linoleic acid <Z>	2124
83.334	2.22	Oleic Acid	2132
88.911	0.82	Eicos-9-en-1-ol <Z>	2259
92.011	0.57	Eicos-11-enoic acid <Z>	2332
95.954	0.44	Docosanal	2429
97.284	0.71	Docos-13-en-1-ol <cis>	2463
108.201	0.47	Benzyl Alkanoate	2755

61.) Basil

RT	Area %	Component	RI
5.66	0.02	Toluene	782
17.45	0.02	1,8-cineole	1030
19.89	0.15	Linalool oxide <cis>	1069
20.947	0.18	Linalool oxide <trans>	1085
21.858	24.99	Linalool	1099
25.145	0.04	Isopulegol	1147
25.656	0.11	Menthone	1154
26.606	0.13	Rosefuran epoxide	1168
27.135	0.64	Menthol	1176
27.375	0.11	Citronella	1180
28.361	0.15	Terpineol <alpha>	1194
28.633	72.4	Methyl chavicol	1198
29.482	0.05	Octyl acetate	1211
31.311	0.08	Neral	1238
33.335	0.09	Geranial	1267
37.503	0.02	Elemene <delta>	1331
40.368	0.02	Copaene <alpha>	1374
43.144	0.1	Caryophyllene <trans>	1419
44.034	0.2	Bergamotene <alpha,trans>	1433
44.533	0.02	Citronellyl propanoate	1441
45.39	0.08	Humulene <alpha>	1455
47.194	0.02	Farnesene	1484
48.697	0.01	Bisabolene <beta>	1509
50.638	0.15	Bisabolene <trans,alpha>	1542
52.264	0.18	Cinnamaldehyde <E,para-methoxy>	1569
52.99	0.04	Caryophyllene oxide	1581

62.) Tulsi

RT	Area %	Component	RI
11.695	0.29	Pinene <alpha>	931
14.091	0.13	Pinene <beta>	976
15.75	0.07	Phellandrene <alpha>	1004
17.155	0.16	Limonene	1026
17.342	1.17	1,8-cineole	1029
21.74	0.58	Linalool	1097
26.049	0.27	Isoborneol	1160
28.457	0.29	Methyl chavicol	1195
30.406	0.95	Citronellol	1224
32.004	1.93	Geraniol	1248
38.407	0.23	Cubebene <alpha>	1344
38.848	44.45	Eugenol	1351
40.274	0.81	Copaene <alpha>	1373
42.117	0.12	Caryophyllene <cis>	1402
42.51	0.09	Isocaryophyllene	1408
43.114	37.62	Caryophyllene <trans>	1418
45.31	6.49	Humulene <alpha>	1453
46.341	0.07	Cadina-1(6),4-diene <cis>	1470
46.892	0.09	Germacrene D	1479
47.99	0.11	Muurolene <alpha>	1497
48.859	0.14	Amorphene <delta>	1512
49.174	0.73	Cadinene <delta>	1517
49.365	0.07	Calamenene <trans>	1520
50.961	1.48	Elemol	1547
52.908	0.8	Caryophyllene oxide	1580
54.53	0.08	Bisabol-11-ol <trans>	1608
55.804	0.1	gamma-Eudesmol	1631
56.388	0.1	Muurolol <alpha>	1641
56.494	0.12	Murrolol <epi,alpha>	1643
57.131	0.46	Cadinol <alpha>	1655

63.) Osmanthus

RT	Area %	Component	RI
17.154	0.32	n-Octanal	1003
17.233	0.42	Hexenyl acetate	1004
21.648	10.33	Linalool oxide <cis>	1070
22.731	8.95	Linalool oxide <trans>	1086
23.621	12.1	Linalool	1099
30.241	0.13	Terpineol <alpha>	1194
32.243	3.16	Nerol	1223
32.403	6.09	Citronellol	1226
34.011	3.08	Geraniol	1249
37.367	1.31	Methyl octine carbonate	1298
38.449	0.1	Theaspirane <trans>	1314
41.757	0.17	Phenethyl alcohol <beta>	1364
43.36	0.4	Elemene <beta>	1388
44.803	0.22	Ionone <dihydro,alpha>	1411
45.597	0.22	Dihydroionene	1423
46.109	20.45	Ionone <dihydro,beta>	1432
46.324	0.32	Citronellyl propionate	1435
46.686	0.84	Ionol <dihydro,beta>	1441
48.149	10.28	Decalactone <gamma>	1464
48.995	18.72	Ionone <trans,beta>	1478
49.158	0.25	Epoxy-beta-Ionone	1480
60.662	0.12	3-Oxo-7,8-dihydro-beta-ionol	1676
62.606	0.25	4-Oxo-7,8-dihydro-beta-ionol	1711
75.299	0.23	Palmitic acid	1956
77.037	0.15	Palmitate <ethyl>	1992
83.507	0.66	Linolenic acid <alpha>	2131
84.674	0.11	Ethyl octadecanoate	2157
84.927	0.4	Ethyl linaloate	2163
105.495	0.11	Tetracosanol	2673

64.) Geranium

RT	Area %	Component	RI
12.974	0.3	Pinene <alpha>	932
23.434	2.11	Linalool	1099
24.158	1.68	Rose oxide <cis>	1110
25.287	0.62	Rose oxide <trans>	1126
27.075	0.15	Citronellal	1151
28.009	5.73	Menthone	1165
32.367	49.69	Citronellol	1228
33.049	0.22	Neral	1238
33.882	6.5	Geraniol	1250
34.797	0.27	Myrtanol <trans>	1263
35.056	0.39	Geranial	1267
35.475	17.86	Citronellyl formate	1273
37.127	1.99	Geranyl formate	1297
37.733	0.29	Terpineol acetate	1306
40.546	0.36	Citronellyl acetate	1348
42.305	0.25	Copaene <alpha>	1375
42.418	0.19	Geranyl acetate	1377
42.834	0.72	Bourbonene <alpha>	1383
45.136	0.66	Caryophyllene <E>	1419
46.372	0.28	Citronellyl propanoate	1439
46.988	0.19	Muuroladiene <cis>	1448
47.665	0.23	Alloaromadendrene	1459
48.985	1.42	Germacrene D	1480
49.594	1.11	Viridiflorene	1490
51.244	0.41	Cadinene <delta>	1517
51.652	0.26	Citronellyl butanoate	1524
55.071	1.04	Phenyl ethyl tiglate	1581
57.417	3.81	Eudesmol <10-epi,gamma>	1622
59.609	0.66	Citronellyl tiglate	1660
61.511	0.61	Geranyl tiglate	1694

65.) Black Spruce

RT	Area %	Component	RI
12.367	0.29	Tricyclene	922
12.512	0.15	Thujene <alpha>	924
12.942	10.06	Pinene <alpha>	932
13.877	4.95	Camphene	948
15.167	1.61	Sabinene	971
15.488	7.13	Pinene <beta>	977
16.115	20.31	Myrcene	988
17.207	0.62	Phellandrene <alpha>	1006
17.357	2.82	Carene <delta-3>	1008
17.88	0.26	Terpinene <alpha>	1016
18.369	0.56	Cymene <para>	1023
18.686	13.21	Limonene	1028
18.797	13.35	Phellandrene <beta>	1030
18.892	1.15	1,8-cineole	1031
20.585	0.4	Terpinene <gamma>	1057
22.423	1.76	Terpinolene	1084
23.38	0.11	Linalool	1098
23.846	4.01	Isopentyl isovalerate	1105
24.503	1.61	Methyl butanoate, 3-methyl-3-butenyl <3>	1115
25.121	0.08	Menth-2-en-1-ol <cis,p>	1123
26.726	10.71	Camphor	1146
27.279	0.16	Terpineol <E,beta>	1154
28.445	0.71	Borneol	1171
29.037	0.5	Terpinen-4-ol	1179
30.044	0.17	Terpineol <alpha>	1194
32.178	0.3	Citronellol	1225
34.068	1.09	Piperitone	1252
36.177	1.5	Bornyl acetate	1283
40.496	0.26	Citronellyl acetate	1348
42.371	0.16	Geranyl acetate	1376

66.) Black Pepper

RT	Area%	Component	RI
12.533	0.75	Thujene <alpha>	925
12.969	12.07	Pinene <alpha>	932
13.894	0.26	Camphene	949
15.202	13.62	Sabinene	972
15.523	14.09	Pinene <beta>	977
16.126	1.03	Myrcene	988
17.23	0.62	Phellandrene <alpha>	1006
17.389	10.14	Carene <delta-3>	1009
17.898	0.22	Terpinene <alpha>	1016
18.388	0.41	Cymene <para>	1024
18.717	14.34	Limonene	1029
18.818	0.93	Phellandrene <beta>	1030
20.602	0.28	Terpinene <gamma>	1057
22.44	0.23	Terpinolene	1084
23.397	0.22	Linalool	1099
29.048	0.16	Terpinen-4-ol	1180
39.597	1.11	Elemene <delta>	1334
40.375	0.13	Cubebene <alpha>	1346
42.272	2.1	Copaene <alpha>	1375
43.057	0.14	Cubebene <beta>	1386
43.153	0.2	Elemene <beta>	1388
45.143	23.35	Caryophyllene <E>	1419
47.355	0.74	Humulene <alpha>	1454
48.939	0.16	Germacrene D	1479
49.838	0.22	Bicyclogermacrene	1494
50.015	0.27	Muurolene <alpha>	1497
50.58	0.79	Bisabolene <beta>	1506
51.202	0.79	Cadinene <delta>	1516
55.012	0.47	Caryophyllene oxide	1580
58.712	0.16	Muurolol <alpha>	1645

67.) Patchouli

RT	Area %	Component	RI
14.079	0.21	Pinene <beta>	976
40.799	1.87	Patchoulene <beta>	1381
41.15	0.85	Elemene <beta,trans>	1387
42.735	0.44	Cycloseychellene	1412
43.032	2.24	Caryophyllene <E>	1417
44.062	10.85	Guaiene <alpha>	1433
44.859	5.42	Seychellene	1446
45.282	0.41	Humulene <alpha>	1453
45.622	3.85	Patchoulene <alpha>	1458
45.804	2.44	Caryophyllene <9-epi>	1461
46.551	0.3	Gurjunene gamma	1473
47.372	0.29	Selinene <beta>	1487
47.803	2.6	Aciphyllene	1494
47.98	0.77	Patchoulene <gamma>	1497
48.183	18.77	Bulnesene <alpha>	1500
49.188	0.21	Selinene <7-epi,alpha>	1517
52.198	0.56	Norpatchoulenol	1568
52.886	0.41	Caryophyllene oxide	1579
53.087	0.43	Spathulenol	1583
54.911	0.29	Atlantol <beta>	1615
55.505	0.72	Citronellyl pentanoate	1626
57.242	2.98	Pogostol	1656
58.006	41.26	Patchouli alcohol	1670
58.112	0.23	Himachalol	1672
60.349	0.63	Pogostone	1713

68.) Tuberose

RT	Area %	Component	RI
17.147	0.23	Limonene	1026
17.337	0.73	1,8-cineole	1029
17.433	2.81	Benzyl Alcohol	1030
21.366	6.13	Methyl benzoate	1091
21.734	1.47	Linalool	1097
22.547	6.83	Phenyl ethyl alcohol	1109
26.031	18.36	Benzyl acetate	1160
27.983	1.34	Methyl salicylate	1189
28.24	3.28	Terpineol <alpha>	1192
37.93	0.57	Anthanilate methyl	1337
38.342	0.56	Benzyl butanoate	1343
38.692	0.59	Eugenol	1349
39.211	0.62	Nonalactone <gamma>	1357
40.496	0.46	Geranyl acetate	1376
41.352	0.79	Jasmone <cis>	1390
41.889	0.98	Methyleugenol	1398
45.247	1.72	Methyl isoeugenol <Z>	1452
47.161	0.61	Jasmolactone <cis>	1483
47.327	3.23	Jasmolactone <trans>	1486
47.878	13.65	Methyl isoeugenol <E>	1495
48.731	0.32	Sesquiterpene	1509
50.531	0.73	Bisabolene	1540
52.331	2.74	Hexenyl Benzoate <3E>	1570
60.483	0.51	Farnesol <2E,6Z>	1716
63.264	18.97	Benzyl benzoate	1767
67.746	0.66	Phenyl-ethyl octanoate	1854
68.477	1.3	Benzyl salicylate	1869
71.434	0.32	Palmitate <methyl>	1929
79.291	0.56	Methyl linoleate	2095
79.643	8.93	Methyl octadecanoate <E>	2103

69.) Douglas Fir

RT	Area %	Component	RI
11.326	0.59	Thujene <alpha>	923
11.72	8.02	Pinene <alpha>	931
12.593	0.34	Camphene	948
13.848	18.37	Sabinene	972
14.147	22.66	Pinene <beta>	978
14.751	2.17	Myrcene	988
15.786	0.17	Phellandrene <alpha>	1004
15.922	9.07	3-Carene	1006
16.427	1.61	Terpinene <alpha>	1014
16.906	0.54	Cymene <para>	1022
17.19	2.26	Limonene	1026
17.291	1.48	Phellandrene <beta>	1028
17.612	0.13	Ocimene <Z,beta>	1033
18.276	0.38	Ocimene <E,beta>	1043
18.68	0.21	2,29-drimethyl-cis-dioxospirononane	1050
19.036	2.96	Terpinene <gamma>	1055
20.817	15.79	Terpinolene	1083
21.143	0.13	Cymenene <para>	1088
21.785	0.14	Linalool	1097
25.36	0.31	Citronellal	1150
27.279	4.65	Terpinen-4-ol	1178
28.295	0.24	Terpineol <alpha>	1193
30.452	1.25	Citronellol	1225
34.335	0.21	Bornyl acetate	1282
38.7	3.7	Citronellyl acetate	1349
40.564	1.74	Geranyl acetate	1377
41.709	0.1	Ethyl decanoate	1396
42.465	0.13	Longifolene	1408
45.363	0.15	Humulene <alpha>	1454
46.957	0.5	Germacrene D	1480

70.) Anthopogon

RT	Area %	Component	RI
12.99	27.65	Pinene <alpha>	933
15.54	14.81	Pinene <beta>	978
16.162	2.65	Myrcene	989
18.736	12.86	Limonene	1029
19.129	7.67	Ocimene <Z,beta>	1035
19.829	1.8	Ocimene <E,beta>	1045
20.645	2.74	Terpinene <gamma>	1057
30.113	0.85	Terpineol <alpha>	1195
42.337	1.42	Copaene <alpha>	1376
45.165	5.24	Caryophyllene <E>	1419
46.342	0.76	Aromadendrene	1438
47.23	1.77	Farnesene <E,beta>	1452
47.42	0.7	Humulene <alpha>	1455
47.688	0.74	Alloaromadendrene	1460
48.446	1.03	Cadina-1(6),4-diene <cis>	1472
48.621	2.24	Cadina-1(6),4-diene <trans>	1474
49.514	2.38	Selinene <beta>	1489
50.084	2.8	Muurolene <alpha>	1498
50.758	0.75	Curcumene <beta>	1509
50.973	2.6	Amorphene <delta>	1513
51.274	6.54	Cadinene <delta>	1518

71.) Rhododendron

RT	Area%	Component	RI
12.557	0.24	Thujene <alpha>	925
13.001	34.53	Pinene <alpha>	933
13.92	0.3	Camphene	949
15.539	14.69	Pinene <beta>	978
16.153	1.94	Myrcene	988
18.416	0.35	Cymene <para>	1024
18.728	8.28	Limonene	1029
19.12	4.88	Ocimene <Z,beta>	1035
19.816	1.02	Ocimene <E,beta>	1045
20.635	3.18	Terpinene <gamma>	1057
22.471	0.54	Terpinolene	1085
23.432	0.46	Linalool	1099
30.091	0.57	Terpineol <alpha>	1195
42.311	0.88	Copaene <alpha>	1375
45.139	2.67	Caryophyllene <E>	1419
47.202	0.67	Farnesene <E,beta>	1452
47.401	0.43	Humulene <alpha>	1455
47.669	0.57	Alloaromadendrene	1459
48.422	0.95	Cadina-1(6),4-diene <cis>	1471
48.598	1.95	Cadina-1(6),4-diene <trans>	1474
49.486	0.43	Selinene <beta>	1488
49.922	1.09	Selinene <alpha>	1495
50.063	2.92	Muurolene <alpha>	1497
50.405	0.34	Farnesene <E,E,alpha>	1503
50.951	3.43	Amorphene <delta>	1512
51.258	10.04	Cadinene <delta>	1517
51.537	0.35	Zonarene	1522
52.353	0.55	Cadinene <alpha>	1536
58.61	0.84	Murrolol <epi,alpha>	1643
59.257	0.91	Cadinol <alpha>	1654

72.) Damask Rose

RT	Area %	Component	RI
12.951	0.19	Pinene <alpha>	932
16.122	0.11	Myrcene	988
23.406	0.62	Linalool	1099
24.195	1.36	Phenyl ethyl alcohol	1110
29.062	0.14	Terpinen-4-ol	1180
30.069	0.17	Terpineol <alpha>	1194
31.473	0.09	Rhodinol	1215
32.078	9.8	Nerol	1223
32.273	31.85	Citronellol	1226
32.425	0.12	Isogeraniol	1228
33.014	0.25	Neral	1237
33.876	20.34	Geraniol	1250
34.096	0.16	2-Phenyl ethyl acetate	1253
35.034	0.45	Geranial	1266
40.617	0.98	Eugenol	1350
42.407	0.34	Geranyl acetate	1377
43.796	1.01	Methyleugenol	1398
45.123	0.2	Caryophyllene <E>	1419
46.101	0.12	Guaiene <alpha>	1434
47.381	0.11	Humulene <alpha>	1455
48.972	0.24	Germacrene D	1480
50.222	0.22	Pentadecane	1500
61.828	2.89	Heptadecane	1700
62.547	0.46	Farnesol <2E,6Z>	1713
70.99	1.26	Nonadec-9-ene	1873
72.355	19.25	Nonadecane	1900
77.227	2.36	Eicosane	1999
81.901	4.47	Heneicosane	2099
83.342	0.08	Oleic Acid	2132
90.657	0.36	Tricosane	2299

73.) Sandalwood

RT	Area%	Component	RI
45.062	1.18	Santalene <alpha>	1418
45.95	0.17	Bergamotene <alpha,trans>	1432
46.838	1.14	Santalene <epi,beta>	1446
47.598	1.79	Santalene <beta>	1458
48.944	0.22	Curcumene	1480
50.707	0.14	Curcumene <beta>	1508
51.214	0.42	Teresantalic acid <alpha>	1517
52.67	0.5	Norekasantalic acid <alpha>	1541
53.341	0.22	Ekasantalic acid <alpha>	1552
59.498	0.17	Santalal	1658
60.008	0.33	Cyclosantalal <epi>	1667
60.321	47.85	Santalol <Z,alpha>	1673
60.51	2.42	Santalol <E,alpha>	1676
61.091	6.6	Bergamotol <Z,alpha,trans>	1687
61.502	0.22	Santalol	1694
61.876	3.55	Santalol <Z,epi,beta>	1700
62.02	0.27	Bergamotol <trans,alpha,trans>	1703
62.195	0.52	Santalol <E,epi,beta>	1706
62.595	23.44	Santalol <Z,beta>	1714
62.976	1.34	Nuciferol <Z>	1721
63.162	0.27	Santalol <epi,beta>	1724
63.419	0.43	Humulene <14-hydroxy,alpha>	1729
63.806	1.53	Santalol <E,beta>	1736
64.035	1.21	Curcumenol	1741
64.14	0.27	Bergamotol	1743
64.638	0.37	Curcumen-12-ol <beta,Z>	1752
64.783	0.68	Ionone <6-methyl>	1754
64.99	1.54	Lanceol <Z>	1758

74.) Winter Savory

RT	Area %	Component	RI
12.522	1.39	Thujene <alpha>	924
12.948	0.97	Pinene <alpha>	932
13.883	0.46	Camphene	948
15.502	1.16	Octen-3-ol	977
16.113	1.86	Myrcene	988
17.214	0.26	Phellandrene <alpha>	1006
17.886	2.48	Terpinene <alpha>	1016
18.385	13.93	Cymene <para>	1024
18.683	0.8	Limonene	1028
18.794	0.21	Phellandrene <beta>	1030
18.894	0.37	1,8-cineole	1031
19.076	0.14	Ocimene <Z,beta>	1034
20.607	19.05	Terpinene <gamma>	1057
21.399	0.71	Sabinene hydrate <cis>	1069
23.384	2.26	Linalool	1099
23.499	0.21	Sabinene hydrate <trans>	1100
26.72	0.22	Camphor	1146
28.445	1.47	Borneol	1171
29.038	0.79	Terpinen-4-ol	1179
30.044	0.36	Terpineol <alpha>	1194
33.779	0.3	Linalyl acetate	1248
37.127	46.01	Carvacrol	1297
41.456	0.31	Carvacryl acetate	1362
45.085	2.42	Caryophyllene <E>	1418
48.538	0.21	Cadina-1(6),4-diene <trans>	1473
48.929	0.2	Germacrene D	1479
50.57	0.79	Bisabolene <beta>	1506
50.891	0.15	Amorphene <delta>	1511
51.188	0.35	Cadinene <delta>	1516
55.002	0.16	Caryophyllene oxide	1580

75.) Tagetes

RT	Area%	Component	RI
9.636	0.11	Trimethylfuran	860
13.09	0.14	Pinene <alpha>	932
14.033	0.13	Camphene	949
15.332	0.69	Sabinene	971
15.652	0.09	Pinene <beta>	977
16.277	0.08	Myrcene	988
17.384	0.27	Phellandrene <alpha>	1006
18.863	6.03	Limonene	1028
19.3	38.77	Ocimene <Z,beta>	1035
19.958	0.45	Ocimene <E,beta>	1045
20.348	25.57	Tagetone <dihydro>	1051
21.812	0.1	Pinene oxide <alpha>	1072
25.537	2.07	Ocimene	1127
25.877	0.35	Epoxyocimene	1132
26.731	0.49	Tagetone <E>	1144
27.22	11.34	Tagetone <cis>	1151
32.682	2.22	Ocimenone <Z>	1230
33.211	3.04	Tagetenone <trans>	1237
45.307	1.08	Caryophyllene <trans>	1419
47.567	0.53	Humulene <alpha>	1455
49.157	0.27	Germacrene D	1480
50.053	1.62	Bicyclogermacrene	1494

76.) Blue Tansy

RT	Area %	Component	RI
11.294	0.31	Thujene <alpha>	923
11.683	3.19	Pinene <alpha>	930
12.556	0.81	Camphene	947
13.804	23.59	Sabinene	971
14.084	7.43	Pinene <beta>	976
14.71	6.31	Myrcene	987
15.735	6.04	Phellandrene <alpha>	1003
16.376	0.81	Terpinene <alpha>	1013
16.854	3.59	Cymene <para>	1021
17.137	2.55	Limonene	1025
17.239	0.39	Phellandrene <beta>	1027
17.329	0.31	1,8-cineole	1028
18.967	1.3	Terpinene <gamma>	1054
20.742	0.45	Terpinolene	1082
24.915	5.93	Camphor	1143
26.614	1.51	Borneol <epi>	1168
27.21	0.91	Terpinen-4-ol	1177
34.674	0.3	Thymol	1287
43.034	1.55	Caryophyllene <trans>	1417
44.402	0.32	Farnesene <Z,beta>	1439
46.87	0.83	Germacrene D	1478
48.727	7.82	3,6-Dihydrochamazulene	1509
48.841	1.23	Dihydrochamazulene A	1511
49.52	0.61	Sesquiphellandrene <beta>	1523
54.753	0.91	Dihydrochamazulene C	1612
55.502	2.18	Dihydrochamazulene D	1626
56.954	1.68	Dihydrochamzaulene E	1651
57.065	0.85	Eudesmol <alpha>	1653
61.156	15.56	Chamazulene	1728
75.282	0.73	Geranyl terpene	2009

77.) Thyme

RT	Area %	Component	RI
6.437	0.07	Methyl-alpha-methyl butyrate	783
12.518	0.16	Thujene <alpha>	924
12.943	0.24	Pinene <alpha>	932
13.879	0.31	Camphene	948
15.169	0.09	Sabinene	971
15.503	0.41	Octen-3-ol	977
16.109	0.34	Myrcene	988
16.554	0.13	3-octanol	995
17.886	0.17	Terpinene <alpha>	1016
18.372	1.69	Cymene <para>	1023
18.679	0.18	Limonene	1028
18.893	0.19	1,8-cineole	1031
20.59	0.61	Terpinene <gamma>	1057
21.401	0.75	Sabinene hydrate <cis>	1069
22.511	0.08	Linalool oxide <trans>	1085
23.441	80.6	Linalool	1099
26.723	0.2	Camphor	1146
28.446	0.22	Borneol	1171
29.041	0.38	Terpinen-4-ol	1180
30.046	0.45	Terpineol <alpha>	1194
32.025	0.19	7-methylene-bicyclo[3.3.1]nonan-3-ol	1223
33.784	5.83	Linalyl acetate	1248
36.5	2.33	Thymol	1288
37.047	0.16	Carvacrol	1296
40.038	0.11	3-Isopropenyl-2-methylenecyclohexyl acetate <Z>	1341
40.314	0.19	Terpinyl acetate <alpha>	1345
42.374	0.68	Geranyl acetate	1376
45.095	2.89	Caryophyllene <E>	1418
49.836	0.09	Bicyclogermacrene	1494
55.014	0.26	Caryophyllene oxide	1580

78.) Damiana

RT	Area%	Component	RI
8.812	0.42	Isovalerate ethyl	846
12.682	5.15	Pinene <alpha>	931
15.206	1.81	Pinene <beta>	976
18.083	1.44	Cymene <para>	1023
18.613	45.67	1,8-cineole	1031
29.666	0.79	Terpineol <alpha>	1193
36.111	0.53	Thymol	1286
39.919	0.91	Cubebene <alpha>	1344
41.84	1.09	Copaene <alpha>	1373
42.734	0.51	Elemene <beta>	1386
44.661	2.62	Caryophyllene <trans>	1416
46.036	1.01	6,9-guaiadiene	1438
47.084	0.64	Velerana-7,11-diene	1455
47.199	0.92	Alloaromadendrene	1457
47.837	3.99	Isolongifolenene <dehydro>	1467
48.138	0.87	Cadina-1(6),4-diene <trans>	1472
48.643	7.84	Epoxy Drimenene	1480
48.9	2.45	Selinene <delta>	1484
49.019	1.14	Selinene <beta>	1486
49.415	1.24	Cubebol <epi>	1492
49.609	1.13	Muurolene <alpha>	1495
49.834	1.93	Dihydroagarofuran <beta>	1499
50.615	0.77	Cadinene <gamma>	1512
50.796	4.15	Cadinene <delta>	1515
53.343	1.7	Nerolidol <trans>	1558
54.593	2.95	Caryophyllene oxide	1579
55.918	0.51	Ledol	1601
56.984	4.42	Selina-6-en-4-ol	1620
64.514	0.61	Valencene <13-hydroxy>	1756

79.) Valerian Root

RT	Area%	Component	RI
12.364	0.39	Tricyclene	922
12.943	7.07	Pinene <alpha>	932
13.779	1.53	Fenchene <alpha>	947
13.896	22.52	Camphene	949
15.165	0.27	Sabinene	971
15.487	4.4	Pinene <beta>	977
18.365	0.26	Cymene <para>	1023
18.673	1.63	Limonene	1028
28.442	1.18	Borneol	1171
30.086	0.27	Myrtenol	1195
32.023	0.25	Thymol <methyl ether>	1223
36.231	41.32	Bornyl acetate	1284
38.758	3.5	Myrtenyl acetate	1321
40.305	0.93	Terpinyl acetate <alpha>	1345
45.081	0.98	Caryophyllene <E>	1418
45.77	0.27	Gurjunene <beta>	1429
47.34	0.36	Humulene <alpha>	1454
47.463	1.54	Aromadendrene <dehydro>	1456
49.435	0.31	Dihydroagarofuran <4-epi,cis>	1487
49.827	0.92	Bicyclogermacrene	1494
51.871	0.67	Kessane	1528
53.554	0.55	3(10)-Caren-4-ol <acetoacetic acid ester>	1556
53.728	0.32	Nerolidol <E>	1559
54.672	1	Spathulenol	1575
59.366	0.21	Intermedeol	1656
63.43	5.79	Isobicyclogermacrenal	1729
66.868	0.61	Bergamotol acetate <Z,alpha,trans>	1793

80.) Vitex

RT	Area%	Component	RI
12.558	0.41	Thujene <alpha>	925
12.989	10.83	Pinene <alpha>	933
15.222	12.53	Sabinene	972
15.534	0.99	Pinene <beta>	977
16.156	1.94	Myrcene	988
17.256	0.56	Phellandrene <alpha>	1007
17.928	0.75	Terpinene <alpha>	1017
18.419	0.56	Cymene <para>	1024
18.732	3.99	Limonene	1029
18.845	2.54	Phellandrene <beta>	1030
18.955	16.48	1,8-cineole	1032
19.82	0.48	Ocimene <E,beta>	1045
20.637	1.23	Terpinene <gamma>	1057
22.475	0.33	Terpinolene	1085
29.095	2.04	Terpinen-4-ol	1180
30.098	0.93	Terpineol <alpha>	1195
39.432	0.31	Elemene <delta>	1332
40.378	7.36	Terpinyl acetate <alpha>	1346
40.557	0.65	Citronellyl acetate	1349
44.359	1.24	Gurjunene <alpha>	1407
45.157	8.67	Caryophyllene <E>	1419
45.983	0.42	Bergamotene <alpha,trans>	1432
46.326	0.28	Aromadendrene	1438
46.457	0.54	Farnesene <Z,beta>	1440
47.227	15.53	Farnesene <E,beta>	1452
47.679	1.82	Alloaromadendrene	1459
48.998	0.66	Germacrene D	1480
49.607	0.28	Viridiflorene	1490
49.897	5.28	Bicyclogermacrene	1495
54.743	0.37	Spathulenol	1576

81.) Plai

RT	Area %	Component	RI
12.679	1.34	Thujene <alpha>	925
13.109	1.43	Pinene <alpha>	933
15.368	21.06	Sabinene	972
15.677	1.95	Pinene <beta>	978
16.298	1.1	Myrcene	989
17.404	0.18	Phellandrene <alpha>	1007
18.081	2.37	Terpinene <alpha>	1017
18.574	3.44	Cymene <para>	1024
18.88	0.48	Limonene	1029
18.993	1.43	Phellandrene <beta>	1031
19.101	0.21	1,8-cineole	1032
20.798	4.39	Terpinene <gamma>	1058
21.614	0.35	4-Thujanol <cis>	1070
22.636	0.89	Terpinolene	1085
23.718	0.26	Sabinene hydrate <trans>	1101
25.346	0.71	Menth-2-en-1-ol <cis,p>	1125
26.589	0.56	Menth-2-en-1-ol <trans,p>	1142
29.312	24.81	Terpinen-4-ol	1181
30.271	0.91	Terpineol <alpha>	1195
31.225	0.4	Piperitol <trans>	1209
40.544	1.28	Terpinyl acetate <alpha>	1346
49.156	1.08	Curcumene <alpha>	1481
50.024	2.2	Zingiberene <alpha>	1495
50.818	0.81	Bisabolene <beta>	1508
51.287	0.78	(3,4-Dimethoxyphenyl)but-1-ene <cis>	1515
51.798	12.62	Sesquiphellandrene <beta>	1524
54.187	0.78	(3',4'-Dimethoxyphenyl)butadiene <Z>	1564
55.685	3.69	(3,4-Dimethoxyphenyl)but-1-ene <trans>	1589
57.856	8.49	(3,4-Dimethoxyphenyl)butadiene <trans>	1627

82.) Ginger

RT	Area%	Component	RI
12.944	2.23	Pinene <alpha>	932
13.885	7.33	Camphene	948
15.486	0.27	Pinene <beta>	977
16.108	0.89	Myrcene	988
17.207	0.2	Phellandrene <alpha>	1006
18.679	1.32	Limonene	1028
18.793	3.84	Phellandrene <beta>	1030
18.891	3.3	1,8-cineole	1031
28.437	0.72	Borneol	1171
30.035	0.37	Terpineol <alpha>	1194
32.97	2.22	Neral	1236
35.002	3.85	Geranial	1266
42.248	0.31	Copaene <alpha>	1374
43.133	0.61	Elemene <beta>	1388
47.15	0.5	Farnesene <E,beta>	1451
48.919	6.92	Curcumene	1479
49.822	34.29	Zingiberene <alpha>	1494
50.038	2.37	Amorphene <gamma>	1497
50.352	5.26	Farnesene <E,E,alpha>	1502
50.578	6.33	Bisabolene <beta>	1506
50.81	0.27	Cadinene <gamma>	1510
51.279	0.5	Panasinsen <(-)-alpha>	1518
51.549	13.2	Sesquiphellandrene <beta>	1522
51.709	0.35	Bisabolene <E,gamma>	1525
53.631	0.41	Germacrene B	1557
53.719	0.45	Nerolidol <E>	1559
56.849	0.53	Zingiberenol	1612
57.818	0.41	7-epi-cis-sesquisabinene hydrate	1629
59.174	0.46	Eudesmol <alpha>	1653
60.907	0.29	7-epi-trans-sesquisabinene hydrate	1683

Supplementary Figure 2. Structures of Potentially Active Major Constituents. All major constituents with potential antifungal activity are shown. Bin numbers are from the similarity analysis.

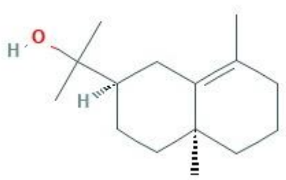

PubChem ID: 6430754
Name: 10-Epi-Gamma-Eudesmol
Bin: 1

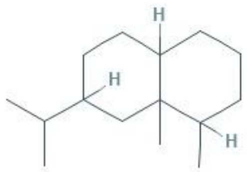

PubChem ID: 27255
Name: 10-Alpha-Eremophilane
Bin: 1

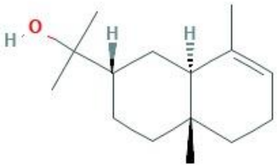

PubChem ID: 12304196
Name: 7-Epi-Alpha-Eudesmol
Bin: 1

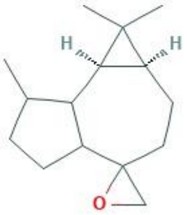

PubChem ID: 91746712
Name: Allo-Aromadendrene Epoxide
Bin: 1

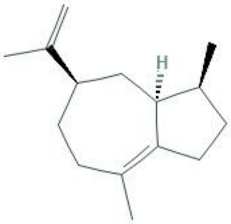

PubChem ID: 94275
Name: Alpha-Bulnesene
Bin: 1

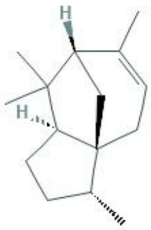

PubChem ID: 6431015
Name: Alpha-Cedrene
Bin: 1

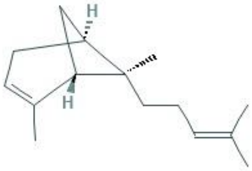

PubChem ID: 6429302
Name: Trans-Alpha-Bergamotol
Bin: 1

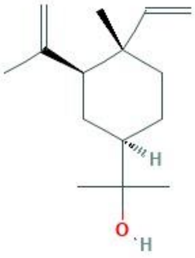

PubChem ID: 92138
Name: Alpha-Elemol
Bin: 1

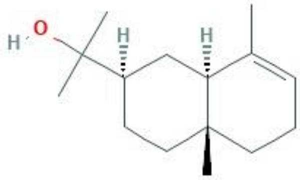

PubChem ID: 92762
Name: Alpha-Eudesmol
Bin: 1

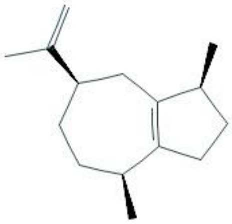

PubChem ID: 5317844
Name: Alpha-Guaiene
Bin: 1

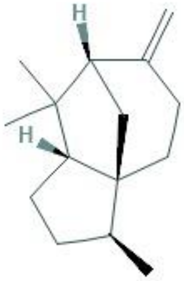

PubChem ID: 60147454
Name: Beta-Funebrene
Bin: 1

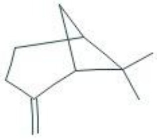

PubChem ID: 14896
Name: Beta-Pinene
Bin: 1

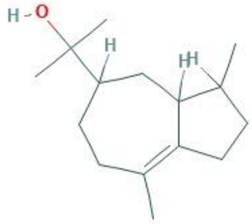

PubChem ID: 90785
Name: Bulnesol
Bin: 1

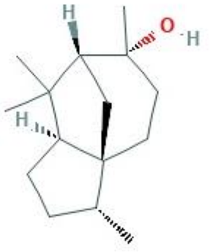

PubChem ID: 65575
Name: Cedrol
Bin: 1

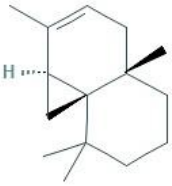

PubChem ID: 442402
Name: Cis-Thujopsene
Bin:1

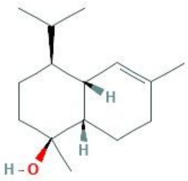

PubChem ID: 3084331
Name: Epi-Alpha-Murrolol
Bin: 1

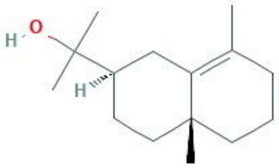

PubChem ID: 6432005
Name: Gamma-Eudesmol
Bin: 1

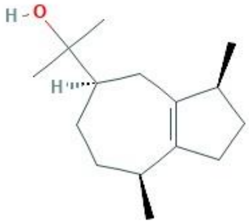

PubChem ID: 227829
Name: Guaiol
Bin: 1

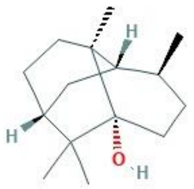

PubChem ID: 10955174
Name: Patchouli Alcohol
Bin: 1

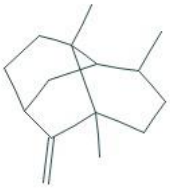

PubChem ID: 519743
Name: Seychellene
Bin: 1

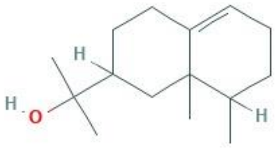

PubChem ID: 146808
Name: Valerianol
Bin: 1

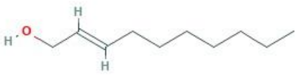

PubChem ID: 5364942
Name: 2-Trans-Decen-1-ol
Bin: 3

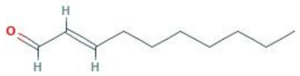

PubChem ID: 5283345
Name: 2-Trans-Decenal
Bin: 3

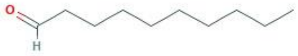

PubChem ID: 8175
Name: n-Decanal
Bin: 3

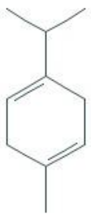

PubChem ID: 7461
Name: Gamma-Terpinene
Bin: 25

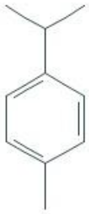

PubChem ID: 7463
Name: Para-Cymene
Bin: 25

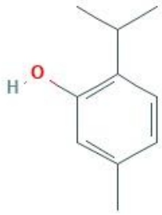

PubChem ID: 6989
Name: Thymol
Bin: 25

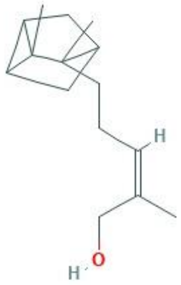

PubChem ID: 5281531
Name: Cis-Alpha-Santalol
Bin: 20

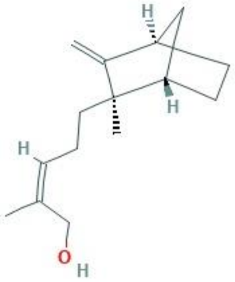

PubChem ID: 6857681
Name: Cis-Beta-Santalol
Bin: 20

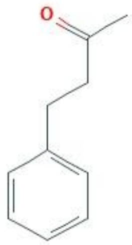

PubChem ID:17355
Name: Benzylacetone
Bin: 13

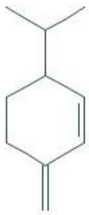

PubChem ID: 11142
Name: Beta-Phellandrene
Bin: 15

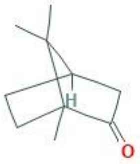

PubChem ID: 2537
Name: Camphor
Bin: 18

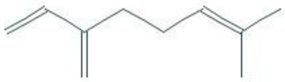

PubChem ID: 31253
Name: Myrcene
Bin: 27
